# Patterns of posttraumatic stress symptoms, their predictors, and comorbid mental health symptoms in traumatized Arabic-speaking people: A latent class analysis

**DOI:** 10.1371/journal.pone.0295999

**Published:** 2023-12-22

**Authors:** Jana Stein, Max Vöhringer, Birgit Wagner, Nadine Stammel, Maria Böttche, Christine Knaevelsrud

**Affiliations:** 1 Clinical Psychological Intervention, Department of Education and Psychology, Freie Universität Berlin, Berlin, Germany; 2 Department for Transcultural and Traumatic Stress Studies, Center ÜBERLEBEN, Berlin, Germany; 3 Clinical Psychology and Psychotherapy, Medical School Berlin, Berlin, Germany; Medical University of Vienna, AUSTRIA

## Abstract

Many people from the Middle East and North Africa (MENA) have experienced traumatic events due to human rights abuses, violence, and conflict in the region, with potential psychological consequences including symptoms of posttraumatic stress and comorbid mental health problems. Yet, little is known about how different posttraumatic stress symptoms unfold in Arabic-speaking people who have experienced diverse traumatic events. This study examined latent classes based on posttraumatic stress symptoms, differences across classes concerning comorbid mental health symptoms and quality of life, and several predictors, including sociodemographic characteristics, social support, and trauma-related characteristics. Participants were 5,140 traumatized Arabic-speaking individuals who had registered for an online intervention. Latent class analysis was conducted to identify distinct classes based on DSM-5 posttraumatic stress symptoms. Multinomial logistic regression was used to analyze predictors of class membership. Differences between classes in severity of posttraumatic stress, depressive, anxiety, and somatoform symptoms, as well as quality of life were examined. Five different latent classes were identified: a general high posttraumatic stress symptom class (43.8%), a high posttraumatic stress symptom-low avoidance class (12.8%), a mixed posttraumatic stress symptom class (20.9%), a high dysphoric-low reexperiencing/avoidance class (14%), and a general low posttraumatic stress symptom class (8.4%). The classes differed in severity of posttraumatic stress, depressive, anxiety and somatoform symptoms, and quality of life. Consistent significant predictors of class membership were gender, social support, cumulative trauma exposure, sexual violence and direct exposure during the most distressing trauma, as well as time since the most distressing trauma. Distinct symptom classes with quantitative and qualitative differences can emerge following exposure to trauma among help-seeking Arabic-speaking people from the MENA region, with gender, social support, and trauma-related characteristics predicting symptom presentation. The results have implications for identifying distressed people and enhancing interventions based on an individual’s symptom presentation.

## Introduction

A large part of the Arabic-speaking population has its roots in the Middle East and North Africa (MENA), a highly populated and culturally diverse geographic area stretching from the Maghrib region in the West to the Mashriq region and the Arabian Peninsula in the East. The MENA region is one of the most severely afflicted by humanitarian crisis situations in the world [1–3]. Political protests, uprisings, and revolutions against totalitarian regimes, which began during the Arab Spring in 2011, have affected nearly all countries located in the region [4]. Countries like Syria, Yemen, Libya, and Iraq continue to suffer from violent armed conflict to this day [1], and in many countries in the MENA region, political imprisonment, executions, or other political murders and brutality as well as unlimited detention may be common [5]. As a result, many civilians have been forced to leave their homes. By the end of 2019, approximately 12.4 million people in the MENA region were still living in internal displacement and 7.8 million had fled abroad [2]. Besides the high rates of forced displacement, political instability, violence, and repression, many countries in the region have been struggling with economic challenges during the past years, including high poverty rates and a lack of future prospects [6].

Exposure to highly stressful life circumstances and traumatic events can impact civilians’ mental health. One of the most prominently researched disorders that can develop in the aftermath of trauma in populations affected by violence, conflict, human rights violations, terror, and political instability is posttraumatic stress disorder (PTSD) [e.g. 7, 8]. The latest edition of the Diagnostic and Statistical Manual of Mental Disorders [DSM-5; 9] lists 20 different posttraumatic stress symptoms (PTSS), including distressing reexperiencing, avoidance of trauma-related stimuli, negative alterations in cognitions and feelings associated with the trauma, and trauma-related alterations in arousal and reactivity.

Pooled PTSD estimates for conflict-affected populations from countries in the MENA region suggest a prevalence rate of approximately 20% to 30%, but rates vary substantially across studies [7, 8]. The use of diagnostic entities for categorization is essential to gain knowledge about the distribution of PTSD in conflict-afflicted countries. However, studies on diagnostic rates do not account for the considerable heterogeneity in posttraumatic stress symptom presentation which can occur in response to trauma. Indeed, Galatzer-Levy and Bryant [10] reported that 636,120 different combinations of DSM-5 posttraumatic stress symptom profiles can emerge. Distinct classes or profiles of posttraumatic stress symptoms can be examined using person-centered approaches. One such approach that has been frequently employed in previous research is latent class analysis (LCA). LCA allows different classes of individuals to be identified based on their response to specific categorical indicators of symptoms [11]. To date, a number of studies have investigated latent posttraumatic stress symptom classes and profiles in traumatized populations originating from non-Western countries that have been affected by violent conflict, terror, displacement, or other human rights abuses in recent years. The majority of these studies used DSM-IV-defined posttraumatic stress symptoms [12–14] or the ICD-11 symptom classification for posttraumatic stress [15–17] as indicators to find latent classes or profiles. Some studies identified latent classes that differed in terms of symptom severity, commonly leading to a three-class solution with different levels of severity [e.g. 13, 18]. Others suggested that posttraumatic stress symptom presentation can also be differentiated by specific, qualitatively distinct latent classes, for example, classes characterized by high affective dysregulation symptoms [15], posttraumatic stress symptoms and low mood [17], or combined symptoms of affective dysregulation, negative self-concept, and interpersonal problems [19]. However, the use of the DSM-5 symptom presentation may yield different findings regarding the characteristics of latent posttraumatic stress symptom classes compared to the DSM-IV or ICD-11. Moreover, the majority of previous studies focused predominantly on posttraumatic stress symptoms with other comorbid mental health complaints, e.g. depression, anxiety and somatization [13, 20], depression and intermittent explosive disorder [21], prolonged grief [14, 22], or complex PTSD [23] as indicators to identify latent patterns of comorbidity or to study discriminant validity.

The choice of a specific set of observed symptom indicators influences the model specification and enumeration process, as LCAs are measurement models [24]. Thus, the use of different combinations of mental health complaints as indicators might lead to different LCA results. To the best of our knowledge, only two studies have focused on the DSM-5 posttraumatic stress symptoms as unique indictors to find latent classes in conflict-affected populations. Minihan, Liddell [25] studied latent classes in 246 adult refugees and asylum seekers from diverse countries living in Australia. A third of the sample (31.7%) was identified as Arabic-speaking. The authors found four classes: a high-PTSD class, a high-reexperiencing/avoidance class, a moderate-PTSD class, and a no PTSD class. Barbieri, Visco-Comandini [26] examined latent posttraumatic stress symptom classes in 122 African refugees and asylum seekers who sought treatment at outpatient units in Italy and found a pervasive class with high probabilities of all symptoms, a moderate class with high avoidance symptoms, and a high-threat class, characterized by high reexperiencing and avoidance symptoms. Besides examining different latent classes based on DSM-5 PTSD symptom endorsement, it is crucial to identify factors that are associated with different symptom classes of posttraumatic stress in order to gain a better understanding of individuals at risk of specific symptoms. Research suggests that sociodemographic characteristics and trauma-related factors are of importance for the development of PTSD [27].

With regard to sociodemographic characteristics, meta-analytic studies on the prevalence of PTSD after human-induced disasters found that gender [27], age [28], level of education [29], the political terror in a country [8], and forced displacement [30] have an impact on the occurrence of posttraumatic stress symptoms. Similar results emerged in studies using LCA. For instance, in a large sample of adults from post-conflict Timor-Leste, Silove, Ivancic [31] reported that gender, employment, area of residence (urban/rural), and level of education were significant sociodemographic covariates of the identified latent classes (using posttraumatic stress symptoms and other mental health complaints as indicators of class membership). However, other studies conducting LCA in participants from conflict-affected non-Western countries, which used DSM-5 posttraumatic stress symptoms as indicators [25, 26], failed to find an association between sociodemographic characteristics and class membership. Another factor that might contribute to posttraumatic stress symptom presentation seems to be social support [29], with research suggesting that individuals with higher perceived social support are more likely to belong to a resilient class than to classes with higher levels of posttraumatic stress symptoms [14].

Concerning trauma-related factors, studies have demonstrated that cumulative exposure to potentially traumatic events and trauma severity constitute important predictors of PTSD in different traumatized populations [e.g. 27]. Furthermore, sexual traumatization has been found to explain a large amount of variance in the severity of posttraumatic stress symptoms, for example, in traumatized West Nile Africans [32]. Additionally, time since primary conflict has emerged as a predictor of PTSD prevalence in populations exposed to conflict and displacement [8]. Moreover, in studies by Minihan, Liddell [25] and Jongedijk, Eising [13], LCA revealed that greater trauma exposure predicted membership of more impaired classes in traumatized refugees. Thus, trauma-related factors might predict distinct profiles of posttraumatic stress symptoms.

Research further suggests that posttraumatic stress symptoms are often associated with other mental health complaints in Arabic-speaking populations affected by conflict and forced displacement [33]. In a population-based survey of Syrian refugees resettled in Sweden, Tinghög, Malm [33] reported high comorbidities between PTSD, depression, and anxiety. Similarly, Renner, Jäckle [34] found that 20% of 133 Syrian refugees in Germany scored above the clinical cutoff for probable mental disorders, namely PTSD and depressive, somatization, and anxiety disorders.

In sum, previous findings suggest that different latent constellations of DSM-5 posttraumatic stress symptoms might develop following traumatic experiences, with different predictors partially explaining the heterogeneity in latent posttraumatic stress symptom presentation. To date, however, no study has investigated the various ways in which traumatized Arabic-speaking people from the MENA region present with DSM-5 posttraumatic stress symptoms. The large sample analyzed in the present study allows us to identify classes of posttraumatic stress symptoms that might have remained undetected in previous studies. Therefore, the current study aimed to 1) identify and describe distinct latent classes based on DSM-5 posttraumatic stress symptom presentation, 2) investigate potential predictors, covering sociodemographic characteristics, social support, and trauma-related factors, of membership of specific latent classes, and 3) examine differences between latent classes with regard to overall posttraumatic stress symptom severity and other mental health complaints (e.g. depressive, anxiety, and somatoform symptom severity) as well as quality of life in a large sample of treatment-seeking Arabic-speaking people from conflict-affected MENA countries. In line with previous research, we expected to identify at least three classes, distinguishable by high, moderate, and low symptomatology. We predicted that sociodemographic characteristics, social support, and trauma-related factors would contribute to the emergence of the latent classes. Finally, we expected that the classes would substantially differ regarding the degree of overall posttraumatic stress, depressive, anxiety and somatoform symptom severity and in terms of quality of life.

## Materials and methods

### Procedure and study population

The current study was part of a larger intervention study. The aim of the intervention study was to investigate the efficacy of online cognitive behavioral treatment in Arabic-speaking people with posttraumatic stress or depressive symptoms [project name: Ilajnafsy (means “psychotherapy” in Arabic); see [35] for a detailed description of the treatment program]. The cognitive behavioral writing treatment has been provided via a secure online platform, and all interactions between counselors and participants have taken place on this platform. Thus, participants for the current study were Arabic-speaking individuals seeking help online for mental health complaints like posttraumatic stress or depression. They were recruited through the treatment program website, network websites (e.g. program blog, social media), and word-of-mouth recommendations. Applicants could register on the program website to gain access to the secure web portal. After providing written informed consent via checkboxes online, they were able to begin the assessment in a password-protected area. Among other aspects, the initial assessment included questionnaires covering demographic data (e.g. age, gender) and questions on trauma exposure and psychopathology (e.g. depression, anxiety, posttraumatic stress symptoms). The data used for the current study served as the baseline measure for participation in the online psychotherapeutic intervention. The intervention study was approved by the Research Ethics Committee of the Freie Universität Berlin. No reimbursement for participation was given.

Inclusion criteria for the present study were as follows: ability to read and write in standard Arabic, age ≥ 18 years, previous exposure to a specific traumatic event, and having completed the PTSD Checklist for DSM-5. Between February 2021 and February 2022, 12,012 participants began with the initial measures online. Of these, 4,843 participants dropped out during the screening process, before providing data regarding traumatic events. Of the remaining 7,169 participants, 1,553 individuals were excluded from further analyses because they did not indicate having experienced a specific traumatic event. Of the 5,616 participants who reported a specific traumatic event, 448 were excluded from the current analyses as they did not provide posttraumatic stress symptom data. A further 27 participants were excluded as they were younger than 18 years old, and one participant was excluded due to invalid data. Thus, the final sample comprised 5,140 participants.

### Assessment

All measures were presented online in a secure web portal. Instruments that were not available in standard Arabic when the study was planned were translated using the following procedure: After the initial translation by a native-speaking individual, the measures were back-translated by a different person who was unaware of the original version. A team of professionals then discussed discrepancies between the two versions and a final version of each assessment instrument was agreed upon. Instruction texts for the original instruments were adapted to fit the online format if necessary.

#### Sociodemographic characteristics

The following sociodemographic characteristics were included in the current study: age, gender, education (no school diploma, intermediate school diploma, high school diploma, university/college diploma), place of residence (metropolitan city, small town, suburb, small village, single farmstead), country of origin, country of residence, and flight experience. Additionally, for the country of origin and the country of residence, the most recent Political Terror Index (PTI) from the Political Terror Scale project [5] was extracted (http://www.politicalterrorscale.org/, use of PTI as indicated by Amnesty International from 2020; PTI range: 1 (secure countries) to 5 (highly insecure countries)).

#### Traumatic events

Exposure to traumatic events was measured using a list combining items from the Harvard Trauma Questionnaire [HTQ; 36], the Posttraumatic Diagnostic Scale [PDS; 37], and the Life Events Checklist for DSM-5 [LEC-5; 38]. In total, the list encompassed 25 items indexing exposure to various potentially traumatizing events (24 specific traumatic events and one “other” category). In line with the DSM-5, participants were asked to affirm that they had been exposed to a traumatic event if it happened to them personally, if they witnessed the event happen to someone else, if they learned about it happening to a close family member/friend, or if they were exposed to it as a part of their job. The sum of affirmative answers for each single event was used to indicate cumulative traumatic events (count of exposure to different traumatic events but without considering the frequency of events). Moreover, participants were asked to provide further details concerning the most distressing traumatic event using items derived from the extended version of the LEC-5, i.e. to whom the event happened, whether someone’s life was in danger, whether the event included sexual violence, and when it happened [38].

#### Posttraumatic stress symptoms

Symptoms of posttraumatic stress in the past month were assessed using the PTSD Checklist for DSM-5 [PCL-5; 39]. The PCL-5 is a self-report questionnaire comprising 20 items that correspond to the DSM-5 PTSD symptoms. Each item is rated on a five-point scale (0 = “not at all”, 1 = “a little bit”, 2 = “moderately”, “3 = quite a bit”, 4 = “extremely”), with higher scores indicating greater symptom severity. For the current analysis, items were dichotomized into a binary response scale and considered as absent if participants responded with 0 or 1 and present if participants responded with 2, 3, or 4, in accordance with the authors’ guidelines [39]. Additionally, a provisional PTSD diagnosis was made following the DSM-5 diagnostic rule that requires the endorsement of at least one item from cluster B (reexperiencing), one item from cluster C (avoidance), two items from cluster D (alteration in cognitions and mood) and two items from cluster E (alterations in arousal and reactivity). A sum score was calculated to assess symptom severity. The PCL-5 has proven to be a valid and reliable screening instrument for traumatized Arabic-speaking populations [40]. In the current sample, Cronbach’s alpha was 0.91.

#### Depressive symptoms

Depressive symptoms in the past two weeks were assessed using the Patient Health Questionnaire-9 [PHQ-9; 41, 42] in an Arabic version [43]. The PHQ-9 comprises nine items covering depressive symptoms, rated on a four-point scale ranging from 0 to 3. A sum score was calculated, with higher scores indicating greater symptom severity. The PHQ-9 has shown good internal consistency in different Arabic-speaking populations [43, 44]. In the current sample, Cronbach’s alpha was 0.82.

#### Anxiety symptoms

Anxiety symptoms in the past two weeks were assessed using the Generalized Anxiety Disorder Scale-7 [GAD-7; 45] in an Arabic version [43]. The questionnaire asks about general anxiety symptoms using seven items rated on a four-point scale ranging from 0 to 3. A sum score was calculated, with higher scores indicating greater symptom severity. While there is evidence to support the reliability and validity of the GAD-7 as a measure of anxiety in Western populations [46], the measure has shown poorer psychometric properties in Arabic-speaking populations [43, 47]. In the current sample, Cronbach’s alpha was 0.83.

#### Somatoform symptoms

Somatoform symptoms in the past four weeks were assessed using the Patient Health Questionnaire-15 [PHQ-15; 48]. The questionnaire includes fifteen somatic symptoms rated on a three-point scale ranging from 0 to 2. A sum score was calculated, with higher scores indicating greater impairment. The PHQ-15 showed good internal consistency in a study with Saudi Arabian university students [44] and was found to be valid in studies conducted with Saudi Arabian primary care patients [47]. In the current sample, Cronbach’s alpha was 0.81.

#### Quality of life

Quality of life was assessed using the EUROHIS-QOL 8-item index, an adapted version of the WHOQOL-100 and the WHOQOL-BREF [49]. The index comprises eight items covering different aspects of quality of life, with items rated on a five-point scale. An overall quality of life sum score was calculated, with higher scores indicating better quality of life. Although an Arabic version of the EUROHIS-QOL 8-item index has not been psychometrically tested, the Arabic version of the WHOQOL-BREF has shown adequate psychometric properties [50]. In the current sample, Cronbach’s alpha was 0.75.

#### Social support

Perceived social support from family, friends, and significant others was measured using the Multidimensional Scale of Perceived Social Support [MSPSS; 51] in a validated Arabic version [52]. The scale comprises 12 items that are rated on a seven-point scale. A sum score was calculated, with higher scores indicating a higher extent of perceived social support. The MSPSS has proven to be a reliable and culturally valid measure in different Arabic-speaking populations [52, 53]. In the current sample, Cronbach’s alpha was 0.90.

### Statistical analysis

A latent class analysis was performed with the Mplus statistical modeling software, version 8 [54]. LCA is a method to identify latent classes of individuals based on responses to a set of items by calculating the probability that an individual belongs to a certain hidden class. The robust maximum likelihood estimator was used for model estimation. To avoid local likelihood solutions, 1,000 random starting value sets were chosen for the initial estimation (with 50 initial stage iterations), with the 100 best starting value sets used for the final stage optimization. Additionally, a small convergence criterion of 0.0000001 was chosen.

To find a model solution that would fit the data best, a series of different latent class models was estimated. First, a one-class model was fitted, and the number of classes was subsequently increased by one to examine whether an additional class led to a better solution until a model with an additional class could not be well identified [55]. To compare models with different numbers of classes, models were evaluated based on statistical and substantive criteria. The most conventional information criteria, namely the Consistent Akaike Information Criterion (CAIC), the Bayesian Information Criterion (BIC), the adjusted Bayesian Information Criterion (aBIC), and the Approximate Weight of Evidence Criterion (AWE), were compared between models [56], with lower values indicating superior fit.

Furthermore, to compare nested models, we examined the results of likelihood-based tests, namely the bootstrapped likelihood ratio test (BLRT) and the Lo-Mendell-Rubin adjusted likelihood ratio test (LMR-LRT) [57, 58]. A significant p-value suggests a statistically significant improvement in fit for the model with one additional class. For the BLRT, 50 bootstrap samples with 500 sets of starting values in the first step and 20 in the second step were specified. It commonly occurs that by adding more classes, the BLRT continues to yield significant p-values and the information criteria indices continue to decrease [55]. In this case, diminishing gains in model fit according to the information criteria (CAIC, BIC, aBIC, AWE) and the log-likelihood (LL) values across models with an increasing number of classes can be explored [55, 59].

Several candidate models were selected based on the statistical model fit results. For these models, classification diagnostics were further examined to evaluate the precision of the latent class assignment for individuals. Entropy as well as the average latent class probability for most likely latent class membership by latent class (ALCP) and odds of correct classification ratio (OCC) were used as diagnostics. Although no definitive conventional cutoff criteria exist, entropy values higher than 0.80 are considered as desirable [60], and ALCP values of higher than 0.70 have been proposed as indicating good class separation and adequate class assignment accuracy [61]. For the OCC, large values for all classes indicate a latent class model with high classification accuracy and good class separation. Nagin [61] suggests an odds ratio value of 5 and above as an indicator of a high degree of assignment accuracy and class separation. Conditional item response probabilities were examined to gain further information on class homogeneity for candidate models [55]. In addition, all candidate models were evaluated regarding substantive as well as theoretical and clinical interpretation. The model with the least number of latent classes with the best combination of model fit indices, classification accuracy, substantive interpretability, and utility of each class was selected as the final optimal class solution. When interpreting the different latent classes, we considered probability values greater than 0.70 of endorsing a specific symptom within each class as high and probability values lower than 0.30 within each class as low [55].

Multinomial logistic regression analysis was performed to assess the impact of predictors on class membership using the three-step approach to account for inaccuracy of class assignment [62, 63]. The following predictors were included: age, gender, education, place of residence (urban/rural), PTI in country of origin, PTI in country of residence, flight experience, social support, cumulative traumatic events, direct exposure to most distressing trauma, involvement of sexual violence during most distressing trauma, danger to someone´s life during most distressing trauma (as an indicator of trauma severity), and time since most distressing trauma. For the multinomial logistic regression, categorical data with more than two categories were dichotomized and dummy coded. Due to the low rate of missing data for auxiliary variables for the predictors PTI and age (n = 30; 0.6%), missing values were subjected to listwise deletion. To evaluate differences in severity of posttraumatic stress, depressive, anxiety, and somatoform symptoms as well as quality of life between classes, we applied the three-step procedure to test for equality of means between classes [e.g. 62, 64].

## Results

### Study population

The sample consisted predominantly of women (76.7%). Participants ranged in age from 18 to 70 years (M = 24.03, SD = 6.79; refers to n = 5,137 due to missing data on age). Participants were highly educated (87.9% had at least a high school diploma). Nearly 90% of the participants were living in urban areas. The largest proportion of participants originated from and were currently residing in Egypt (28.3% and 29.6%, respectively) and Saudi Arabia (25.2% and 24.3%, respectively) (see Table 1 for further information).

**Table 1:** Sociodemographic information of participants (N = 5,140).

	n (%)
Female gender	3,943 (76.7)
Education	
Low education (no or intermediate school diploma)	620 (12.1)
High education (high school or university/college diploma)	4,520 (87.9)
Place of residence	
Urban (metropolitan city, small town or suburb)	4,586 (89.2)
Rural (small village or single farmstead)	554 (10.8)
Flight experience	406 (7.9)
Country of origin	
Egypt	1,454 (28.3)
Saudi Arabia	1,294 (25.2)
Syria	341 (6.6)
Jordan	315 (6.1)
Morocco	268 (5.2)
Iraq	265 (5.2)
Others (e.g. Algeria, Tunisia)	1,203 (23.4)
Political Terror Index (country of origin)^a^	
Low Political Terror Index (max. score 2)	917 (17.8)
High Political Terror Index (min. score 3)	4,206 (81.8)
Country of residence	
Egypt	1,524 (29.6)
Saudi Arabia	1,250 (24.3)
Jordan	329 (6.4)
Morocco	244 (4.7)
Iraq	228 (4.4)
Others (e.g. Algeria, Tunisia)	1,565 (30.4)
Political Terror Index (country of residence)^a^	
Low Political Terror Index (max. score 2)	1,135 (22.1)
High Political Terror Index (min. score 3)	3,988 (77.6)

^a^Number of participants reduced to *n* = 5,123 due to missing information on the Political Terror Index of the specific country; The Political Terror Index (Amnesty International Score) seeks to measure political terror on a 5-point ordinal scale, with a score of 1 indicating the minimum level of political terror and a score of 5 indicating the maximum level; n = sample size.

Participants reported having been exposed to between 1 and 24 different traumatic events from the list provided (M = 4.64, SD = 3.42). The frequencies of reported specific traumatic events are listed in Table 2. The most frequently reported specific most distressing traumatic events (excluding the “other” category) were sexual assault under the age of 18 (12.3%), sexual assault by a family member or acquaintance (8.1%), and unnatural death of a family member or friend (5.5%). In line with this, 19.4% of the participants reported an involvement of sexual violence during the most distressing event. The majority of participants experienced the most distressing traumatic event either personally or as a witness (80.4%) and reported that this event took place more than three years ago (57.2%) (see Table 2).

**Table 2:** List of traumatic events and information on most distressing event reported by participants (N = 5,140).

	n (%)
Frequency of exposure to specific traumatic events^a^	
Sexual contact while under the age of 18 with a person at least 5 years older (e.g. contact with genitals or breasts)	2,470 (48.1)
Being close to death	1,814 (35.3)
Poor health without access to medical care	1,724 (33.5)
Lack of food or water	1,227 (23.9)
Life-threatening illness	1,214 (23.6)
Sexual assault by a family member or acquaintance (e.g. rape or attempted rape)	1,152 (22.4)
Serious accident, fire, or explosion (e.g. industrial accident, agricultural accident, car accident, airplane or ship accident)	1,140 (22.2)
Unnatural death of a family member or friend	1,115 (21.7)
Sexual assault by stranger (e.g. rape or attempted rape)	1,016 (19.8)
Violent attack by a family member or acquaintance (e.g. being physically attacked, robbed, shot at or threatened with a firearm, stabbed)	1,000 (19.5)
Serious injury	818 (15.9)
Combat deployment in war or stay in war zone	733 (14.3)
Violent assault by stranger (e.g. being physically assaulted, robbed, shot at or threatened with a firearm, stabbed)	684 (13.3)
Forced separation from family members	622 (12.1)
Murder of a stranger or strangers	572 (11.1)
Murder of a family member or friend	530 (10.3)
Natural disaster (e.g. hurricane, tornado, flood disaster, severe earthquake)	526 (10.2)
Torture	509 (9.9)
Forced isolation	474 (9.2)
Not having a roof over one’s head	437 (8.5)
Captivity (e.g. penal prisoner, prisoner of war, hostage)	335 (6.5)
Disappearance or kidnapping	332 (6.5)
Brainwashing	313 (6.1)
Serious injury, damage, or death caused to someone else by participant	278 (5.4)
Sexual violence during most distressing trauma	998 (19.4)
Direct exposure to most distressing trauma (happened directly or witnessed it)	4,130 (80.4)
Danger to someone´s life during most distressing trauma	2,472 (48.1)
Time since most distressing trauma	
Less than three years ago	2,198 (42.8)
More than three years ago	2,942 (57.2)

^a^Multiple answers for traumatic event possible; n = sample size.

Of the overall sample, 67.7% (n = 3,482) met criteria for probable PTSD according to the DSM-5, and the average symptom endorsement for other measures of psychopathology was high (see Table 3).

**Table 3:** Information on psychopathology, quality of life and social support of participants (N = 5,140).

	M	SD	Range
Posttraumatic stress symptom severity (PCL-5)	47.49	16.43	0–80
Depressive symptom severity (PHQ-9)	18.75	5.63	0–27
Anxiety symptom severity (GAD-7)	15.16	4.67	0–21
Somatoform symptom severity (PHQ-15)	15.36	5.48	0–30
Quality of life (EUROHIS-QOL-8)	11.78	5.28	0–32
Social support (MSPSS*)*	39.14	17.24	12–84

PCL-5 = PTSD Checklist for DSM-5 (Diagnostic and Statistical Manual of Mental Disorders, version 5); PHQ-9 = Patient Health Questionnaire-9; GAD-7 = Generalized Anxiety Disorder Scale-7; PHQ-15 = Patient Health Questionnaire-15; EUROHIS-QOL-8 = EUROHIS Quality of Life 8-item index; MSPSS = Multidimensional Scale of Perceived Social Support; M = Mean; SD = Standard Deviation.

### Latent class analysis

#### Selecting the optimal class solution

Unconditional latent class models with one to eight classes were fitted using binary response indicators (with a value of 1 indicating symptom endorsement) for the DSM-5 posttraumatic stress symptom items. Models with one to seven classes appeared to be well identified. For the 8-class model, boundaries emerged, indicating the extraction of too many classes. Therefore, no additional models with more than eight classes were fitted. The results of the latent class analyses for all tested models are summarized in Table 4.

**Table 4:** Goodness-of-fit information for different latent classes (N = 5,140).

Model	npar	LL	CAIC	BIC	aBIC	AWE	LMR-LRT p-value	BLRT p-value	Entrophy	Lowest class-specific ALCP	Lowest class-specific OCC	Sample size per class based on most likely class membership
1-class	20	-58609	117409	117388	117325	117620	-	-	-	-	-	5140
2-class	41	-50571	101533	101492	101362	101965	<0.001	<0.001	0.89	0.95	17.93	3487,1653
3-class	62	-49016	98624	98562	98365	99278	<0.001	<0.001	0.84	0.91	14.49	2708,1879,553
4-class	83	-48170	97133	97050	96786	98008	<0.001	<0.001	0.83	0.84	14.67	2739,780,1146,475
**5-class**	**104**	**-47570**	**96132**	**96028**	**95698**	**97229**	**<0.001**	**<0.001**	**0.83**	**0.84**	**16.60**	**2250,660,1076,433, 721**
6-class	125	-47181	95556	95431	95034	96874	0.018	<0.001	0.80	0.77	13.94	639,700,987,638, 347,1829
7-class	146	-46935	95264	95118	94654	96803	0.014	<0.001	0.78	0.75	16.50	1601,641,790,545, 531,742,290
8-class^a^	167	-46775	95143	94976	94446	96904	0.107	<0.001	0.79	0.75	16.01	1645,235,651,457, 807,650,433,262

^a^Class solution includes boundary estimates; npar = number of parameters; LL = Log-Likelihood; CAIC = Consistent Akaike Information Criterion; BIC = Bayesian Information Criterion; aBIC = adjusted Bayesian Information Criterion; AWE = Approximate Weight of Evidence Criterion; LMR-LRT = Lo-Mendell-Rubin adjusted likelihood ratio test; BLRT = bootstrapped likelihood ratio test; ALCP = average latent class probability; OCC = odds of correct classification ratio; Most meaningful model is printed in **bold**.

The lowest AWE was for the 7-class solution. The CAIC, BIC, and aBIC decreased for all models. The BLRT revealed significant results for all model comparisons. Accordingly, there was no “best” model solution to be selected based on the CAIC, BIC, aBIC and the BLRT. An exploration of gain in model fit showed that the CAIC, BIC, aBIC as well as the LL decreased substantially when moving from a model with one to two latent classes and from a model with two to three latent classes. Subsequent models with additional classes showed a diminished gain in the CAIC, BIC, aBIC, and LL. Due to the large gain in model fit up to the 3-class solution, the 1-class and 2-class solutions were discarded (see S1 Fig for gain in fit indices across all models). The LMR-LRT showed non-significant results for the model with eight classes compared to the 7-class model, indicating that the 8-class solution did not fit the data better than the models with fewer classes. As boundaries emerged, the LMR-LRT showed non-significant results, and the gain in model fit (CAIC, BIC, aBIC, LL) was low for the addition of an eighth class; this class solution was discarded.

The results of the model fit indices hint at an optimal solution for a model with three to seven classes. Therefore, we further examined classification diagnostics as well as substantive meaning and clinical utility of the classes for each of the models. Regarding classification accuracy, the overall entropy was above 0.80 for models with one to six classes and 0.78 for the model with seven classes. The lowest class-specific ALCP of each model was above 0.70 for all class solutions. The lowest class-specific OCC value was equal to or greater than 5 for all model solutions. In all model solutions, one class represented a clear profile with a high probability of endorsing all symptom items with the exception of inability to remember important parts of the trauma, and a profile with a low probability of endorsing nearly all symptom items. The high and the low symptom classes were well separated from each other in all model solutions. In each solution, one class remained heterogenous regarding the probability of endorsing at least over half of the symptom items (see S2 Fig for profile plots for models with three to seven classes). Compared to the 3-class model, the 4-class model revealed a unique interpretable symptom class with a substantial percentage of participants (15.2%), characterized by a high probability of endorsing most of the symptom items representing negative alterations in cognition and mood, irritability and aggression, difficulties in concentrating, and sleep disturbances. This class showed a low probability of endorsing most of the reexperiencing symptom items and avoidance of external reminders. The 5-class model revealed a uniquely interpretable additional symptom class with 12.8% of all participants characterized by a low probability of endorsing both avoidance symptom items and the inability to remember important parts of the trauma, and by a high probability of endorsing nearly all other symptom items. Compared to the 5-class model, the 6-class model revealed an additional class (19.2%) with a low probability of endorsing the presence of nightmares and a high probability of endorsing all other symptom items except for dissociative reactions, physical reactivity to reminders, inability to remember important parts of the trauma, and risky behavior. For these latter symptom items, the probability of endorsement was neither high nor low. A similar class emerged in the 7-class model (with 15.4% of participants), but with a low probability of endorsing nightmares and dissociative reactions and a high probability of endorsing all other symptom items except for intrusive memories, physical reactivity to reminders, inability to remember important parts of the trauma, and risky behavior. For these latter symptom items, the probability of endorsement was neither high nor low. In addition, the 7-class model revealed a class (14.4%) characterized by a high probability of endorsing all symptom items except for irritability and aggression, risky behavior, hypervigilance, easily startled, inability to remember important parts of the trauma, and nightmares. For these latter symptom items, the probability of endorsement was neither high nor low. The additional classes that emerged in the 6-class and the 7-class model were very similar to the class with a high probability of endorsing all symptom items found in all class solutions and were therefore deemed redundant. Thus, the 6- and the 7-class model seemed to extract too many classes, rendering it difficult to interpret specific classes, and showed a low separation between numerous classes in both solutions. In contrast, the 3-class and 4-class models, which showed an overall good class separation, seemed to cover too much heterogeneity in the population. Therefore, the 5-class solution was selected as the final model given its good fit indices, classification precision, and substantive interpretability. An overview of classification diagnostics for the 5-class solution is displayed in Table 5.

**Table 5:** Model classification diagnostics for the 5-class solution (N = 5,140).

Classes	Estimated class proportion	ALCP	OCC	Entropy
Class 1—General high PTSS class	0.43	0.93	16.60	0.83
Class 2—High PTSS-low avoidance class	0.13	0.84	35.89	
Class 3—Mixed PTSS class	0.21	0.85	20.46	
Class 4—High dysphoric-low reexperiencing/ avoidance class	0.14	0.87	40.11	
Class 5—General low PTSS class	0.09	0.94	165.51	

PTSS = Posttraumatic stress symptoms; ALCP = average latent class probability for a specific class; OCC = odds of correct classification ratio for a specific class.

#### Description of latent classes

**Fig 1:**
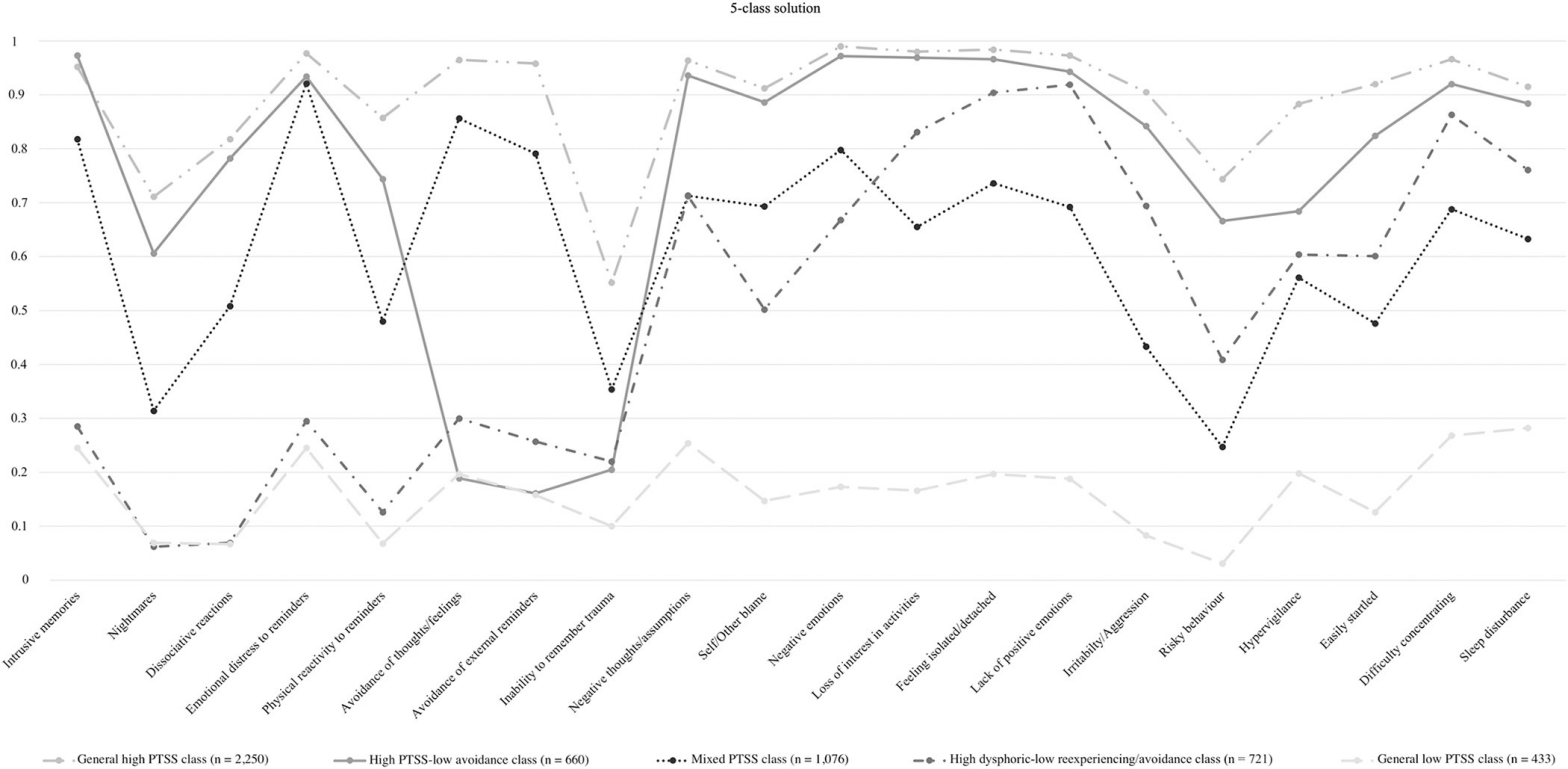
Class-specific item response probability profile plots for symptom endorsement for the 5-class solution.

Fig 1 shows the profile plot with the estimated class-specific symptom item response probabilities for the 5-class solution. The classes within this solution can be labeled as the general high PTSS class (n = 2,250; 43.8%), the high PTSS-low avoidance class (n = 660; 12.8%), the mixed PTSS class (n = 1,076, 20.9%), the high dysphoric-low reexperiencing/avoidance class (n = 721; 14%), and the general low PTSS class (n = 433; 8.4%). Rates of probable PTSD diagnosis according to class membership were as follows: general high PTSS class: n = 2,250 (100%), high PTSS-low avoidance class: n = 190 (28.8%), mixed PTSS class: n = 854 (79.4%), high dysphoric-low reexperiencing/avoidance class: n = 179 (24.8%), and general low PTSS class: n = 9 (2.1%). Participants assigned to the general high PTSS class showed a high probability of endorsing all symptoms except for the inability to remember important parts of the trauma. Participants assigned to the high PTSS-low avoidance class showed a low probability of endorsing both avoidance symptom items and the inability to remember important parts of the trauma. For nearly all other symptom items, the probability of endorsement was high. This class had a high level of homogeneity with respect to all symptom items except for nightmares, risky behavior, and hypervigilance, meaning that for these three symptom items, it was not characterized by either high or low response probabilities. Participants assigned to the mixed PTSS class showed a high probability of endorsing intrusive memories, emotional distress due to reminders, avoidance of thoughts and feelings, avoidance of external reminders, negative thoughts and assumptions, negative emotions, and feeling isolated/detached, and a low probability of endorsing the risky behavior item. This mixed PTSS class did not show a high level of homogeneity with respect to the other symptom items, meaning that it was characterized neither by high nor low endorsement probabilities regarding these symptom items. Participants assigned to the high dysphoric-low reexperiencing/avoidance class showed a low probability of endorsing intrusive memories, nightmares, dissociative reactions, emotional distress due to reminders, physical reactivity to reminders, avoidance of external reminders, and inability to remember important parts of the trauma. The probability of endorsing avoidance of thoughts and feelings was marginally above the cutoff (value of precisely 0.30) and can likewise be considered low. Participants in this high dysphoric-low reexperiencing/avoidance class showed a high probability of endorsing negative thoughts and assumptions, negative emotions, loss of interest in activities, feeling isolated/detached, lack of positive emotions, difficulty concentrating, and sleep disturbances. For the other symptom items, it was characterized by neither high nor low endorsement probabilities. Finally, participants assigned to the general low PTSS class showed a low probability of endorsing all symptom items and showed a high level of homogeneity with respect to all symptom items. Item response probabilities of symptom endorsement and standard errors are displayed for each class separately in S1 Table.

### Multinomial logistic regression

Several predictors of class membership emerged as significant (see S2 Table). The Bonferroni correction was applied to account for multiple testing and keep the error rate at 0.05. Thus, a p value of < 0.005 (adjusted for ten class comparisons) indicated statistical significance.

#### Sociodemographic characteristics

Compared to the general low PTSS class, males were less likely to be in the general high PTSS class, the high PTSS-low avoidance class, and the mixed PTSS class (p < 0.001, p = 0.005 for the high PTSS-low avoidance class). Of the symptomatically burdened classes, males were most likely to be in the high dysphoric-low reexperiencing/avoidance class (p ≤ 0.001).

#### Trauma characteristics

Compared to the general low PTSS class, the likelihood of belonging to any symptomatically burdened class was higher in participants with higher levels of cumulative trauma (p = 0.002 for the high dysphoric-low reexperiencing/avoidance class, p < 0.001 for all other classes). Relative to the general low PTSS class, the likelihood of belonging to the general high PTSS class and the mixed PTSS class was higher in participants who experienced sexual violence during the most distressing event (p < 0.001). Moreover, compared to the general low PTSS class, the likelihood of belonging to the general high and the mixed PTSS class was higher in participants who experienced the most distressing event directly (p = 0.003 and p = 0.001, respectively). The likelihood of membership in the general high PTSS and the high PTSS-low avoidance class, as compared to the general low PTSS class, was higher in participants who experienced the most distressing event less than three years ago (p < 0.001).

Compared to the general high PTSS class and the high PTSS-low avoidance class, participants with a higher level of cumulative trauma were less likely to belong to the mixed PTSS class and to the high dysphoric-low reexperiencing/avoidance class (p < 0.001).

The likelihood of membership in the high dysphoric-low reexperiencing/avoidance class, as compared to the general high PTSS class, was lower in participants who experienced sexual violence during the most distressing event (p < 0.001). Likewise, these individuals were less likely to belong to the high dysphoric-low reexperiencing/avoidance class than to the mixed PTSS class (p < 0.001).

Compared to the general high PTSS class, the likelihood of membership in the high PTSS-low avoidance class was lower in participants who were directly exposed to the most distressing event (p = 0.002). These participants were more likely to belong to the mixed PTSS class than to the high PTSS-low avoidance class (p = 0.001).

The likelihood of membership in the high PTSS-low avoidance class, as compared to the general high PTSS class, was higher in participants who experienced the most distressing event less than three years ago (p = 0.003). Compared to the general high PTSS and the high PTSS-low avoidance classes, the likelihood of belonging to the mixed PTSS class and the high dysphoric-low reexperiencing/avoidance class was lower in participants who experienced the most distressing event less than three years ago (p < 0.001).

#### Social support

Compared to the general low PTSS class, the likelihood of membership in the general high PTSS class, the high PTSS-low avoidance class, and the high dysphoric-low reexperiencing/avoidance class was lower in participants with higher social support (p < 0.001). Similarly, participants with lower social support were more likely to belong to the high dysphoric-low reexperiencing/avoidance class than to the mixed PTSS class (p < 0.001). Compared to the high PTSS class and the high PTSS-low avoidance class, the likelihood of belonging to the mixed PTSS class was higher in people with higher social support (p < 0.001).

### Differences in symptom severity and quality of life between classes

The Bonferroni correction was applied and a p value of < 0.005 (error rate kept at 0.05, adjusted for ten comparisons) indicated statistical significance. The overall test for equality of means using the three-step approach indicated significant differences between the classes for overall posttraumatic stress symptom severity (χ2(4) = 13244.15, p < 0.001), with the general high PTSS class showing the highest mean value (M = 61.31), followed by the high PTSS-low avoidance class (M = 51.56), the mixed PTSS class (M = 39.85), the high dysphoric-low reexperiencing/avoidance class (M = 34.11), and finally the general low PTSS class (M = 12.65). All pairwise comparisons were significant at p ≤ 0.001. With regard to other mental health complaints and quality of life, the overall test for equality of means using the three-step approach indicated significant differences between the classes regarding depressive symptom severity (χ2(4) = 1157.37, p < 0.001), somatoform symptom severity (χ2(4) = 830.16, p < 0.001), anxiety symptom severity (χ2(4) = 1012.83, p < 0.001), and quality of life (χ2(4) = 554.39, p < 0.001). For depressive symptom severity, the general high PTSS class showed the highest mean value (M = 21.15), followed by the high PTSS-low avoidance class (M = 20.47), the high dysphoric-low reexperiencing/avoidance class (M = 18.76), the mixed PTSS class (M = 15.16), and the general low PTSS class (M = 13.04). The comparison between the general high PTSS class and the high PTSS-low avoidance class was not significant after Bonferroni correction (p = 0.007). All other pairwise comparisons were significant at p ≤ 0.001. For somatoform symptom severity, the general high PTSS class showed the highest mean value (M = 17.40), followed by the high PTSS-low avoidance class (M = 16.79), the high dysphoric-low reexperiencing/avoidance class (M = 14.05), the mixed PTSS class (M = 12.95), and the general low PTSS class (M = 11.06). The comparison between the general high PTSS class and the high PTSS-low avoidance class was not significant after Bonferroni correction (p = 0.029). All other pairwise comparisons were significant at p ≤ 0.001. For anxiety symptom severity, the general high PTSS class showed the highest mean (M = 17.08), followed by the high PTSS-low avoidance class (M = 16.64), the high dysphoric-low reexperiencing/avoidance class (M = 14.53), the mixed PTSS class (M = 12.58), and the general low PTSS class (M = 10.80). The comparison between the general high PTSS class and the high PTSS-low avoidance class was not significant after Bonferroni correction (p = 0.035). All other pairwise comparisons were significant at p ≤ 0.001. For quality of life, the general low PTSS class showed the highest mean (M = 15.26), followed by the mixed PTSS class (M = 14.45), the high dysphoric-low reexperiencing/avoidance class (M = 11.58), the general high PTSS class (M = 10.34), and the high PTSS-low avoidance class (M = 10.12). The comparison between the general high PTSS class and the high PTSS-low avoidance class as well as between the mixed PTSS class and the general low PTSS class was not significant (p = 0.382 and p = 0.017, respectively). All other pairwise comparisons were significant at p ≤ 0.001.

## Discussion

The purpose of the present study was to explore distinct latent classes based on DSM-5 posttraumatic stress symptoms in a large sample of Arabic-speaking people from the MENA region who had been exposed to diverse traumatic events and sought help for mental health problems. Furthermore, we examined the predictive impact of sociodemographic and trauma-related characteristics and of social support on class membership, and analyzed the differences between the classes with regard to symptom severity of numerous mental health complaints and quality of life. The results reveal that exposure to traumatic events can lead to different combinations of DSM-5 posttraumatic stress symptom presentation. The latent class analysis pointed to a 5-class solution: a general high PTSS class, a high PTSS-low avoidance class, a mixed PTSS class, a high dysphoric-low reexperiencing/avoidance class, and a general low PTSS class. All classes differed significantly from each other regarding overall posttraumatic stress, anxiety, depressive and somatoform symptom severity. The most consistent predictors of class membership were gender, social support, and different trauma-related aspects like cumulative trauma exposure.

Numerous previous studies examining similar populations identified a general high PTSS, a mixed/moderate PTSS, and a general low/no PTSS class [e.g. 25, 65], whereas the identification of a high PTSS-low avoidance class and a high dysphoric-low reexperiencing/avoidance class in the present study represents a novel finding. In our study, the general high PTSS class had a high probability of experiencing all but one posttraumatic stress symptom. As such, this class, which included the largest proportion of participants (43.8%), showed the highest mean posttraumatic stress symptom severity, and all participants in this class met criteria for probable PTSD. Furthermore, the participants in this class showed higher symptom severity of mental health complaints than did participants assigned to other classes. It is not surprising that the general high PTSS class included the largest proportion of participants with high levels of symptom severity, given that our sample consists of highly distressed individuals seeking help for mental health problems online.

The high PTSS-low avoidance class, which encompassed 12.8% of the participants, was characterized by a low probability of both avoidance symptoms and a high probability of nearly all other symptoms. Posttraumatic stress symptom severity was significantly lower compared to the general high PTSS class but comorbid symptomatology like depressive, anxiety and somatoform symptom severity were similar to the general high PTSS class. This finding demonstrates that individuals assigned to the general high PTSS class or the high PTSS-low avoidance class experienced a variety of comorbid mental health symptoms, in line with previous research reporting high comorbidities between mental health symptoms in traumatized Arabic-speaking populations [66]. Even though the high PTSS-low avoidance class was similar to the general high PTSS class in terms of symptom distress and disturbance, 71.2% of participants in this class did not meet criteria for probable PTSD. The occurrence of a class characterized by the absence of avoidance may be related to the characteristics of our sample: Studies with traumatized Arabic-speaking participants have reported lower rates of individuals meeting the avoidance symptom criteria compared to the reexperiencing and hyperarousal criteria [67, 68]. These studies used DSM-IV-defined PTSD, which grouped together symptoms of avoidance, emotional numbing, memory loss as well as lack of future plans, loss of interest and feeling distant. Nevertheless, the study by Norris and Aroian [67] with traumatized Arabic-speaking women who had immigrated to the United States found that the reported frequency of avoiding places, people and activities was lower than the reported frequency for any of the reexperiencing and hyperarousal symptoms. This suggests a subgroup of traumatized Arabic-speaking individuals who exhibit cardinal posttraumatic stress symptoms without showing active avoidance. Individuals in the high PTSS-low avoidance class appear to have a high level of distress due the symptom severity of the reexperience, negative thoughts/feelings and hyperarousal cluster, but would most probably not fulfil the criteria for PTSD. Nevertheless, it seems to be important to determine whether low-threshold psychosocial support in the sense of a stepped-care approach is required for this class in order to reduce the existing posttraumatic stress symptoms and possibly prevent the development of mental disorders.

The high dysphoric-low reexperiencing/avoidance class, which included 14% of all participants, showed high probabilities for key symptoms typical of depression (i.e. loss of interest in activities, lack of positive emotions) and low probabilities for symptoms that reflect the core learned fear response that is typical for PTSD. Participants in this class showed a significantly lower mean posttraumatic stress symptom severity than the other symptomatically burdened classes but they experienced greater depressive symptom severity and a lower quality of life than the classes with general low or mixed PTSS. To the best of our knowledge, a high dysphoric-low reexperiencing/avoidance class has not been previously identified in LCA studies examining similar populations and using DSM-5 measures of posttraumatic stress symptoms. Using DSM-5 measures of posttraumatic stress symptoms, some studies examined typologies of PTSD in U.S. samples (e.g. military personnel) [69, 70]. Similar to our findings, these studies also revealed a class characterized by symptoms of anhedonia and negative affect (commonly referred to as ‘dysphoric’). In contrast to the high dysphoric-low reexperiencing/avoidance class found in our sample which was characterized by low probability of endorsing any reexperiencing and avoidance symptoms, the dysphoric class in the other studies was characterized by medium to high probabilities of endorsing some reexperiencing (like intrusive thoughts) and/or avoidance symptoms [69, 70]. Further, numerous studies using other methods have reported that a depressive/dysphoric reaction, without exhibiting posttraumatic stress symptoms, is common among traumatized Arabic-speaking populations [e.g. 33, 66]. Moreover, in a study by Tay, Rees [21] in a sample of West Papuan refugees, LCA using item responses to culturally adapted measures of depression, intermittent explosive disorder, and posttraumatic stress as indicators yielded a class of people who reported predominantly depressive symptoms and moderate levels of endorsement for most DSM-IV posttraumatic stress symptoms. In a sample of refugees in Australia, a measure that specifically captured depressive and anxiety symptoms (besides symptoms of posttraumatic stress) for latent classes, a class with high depressive/anxiety symptoms but without posttraumatic stress symptoms was identified [65].

In the current study, only a small proportion of our participants (8.4%) belonged to the general low PTSS class, which was characterized by a low probability of all posttraumatic stress symptoms. Members of this class had the lowest levels of posttraumatic stress, depressive, anxiety, and somatoform symptom severity. Although previous research with similar populations found a general high, a mixed/moderate, and a general low/no PTSS class, these studies reported lower rates of participants assigned to the general high PTSS class and higher rates of participants assigned to the general low PTSS class [12, 21, 25].

In contrast to studies reporting an intermediate symptom class characterized by neither high nor low probabilities of symptom endorsement [25], in the present study, the mixed PTSS class with moderate to high probabilities of symptom endorsement showed high probabilities for numerous symptom items. This class comprised one fifth of all participants. Therefore, a higher number of our Arabic-speaking participants seem to endorse more posttraumatic stress symptoms overall as compared to other studies, which is potentially attributable to the specifics of the current sample. Research suggests that the burden of mental disorders in the MENA region is higher than the global level [71], while the mental health structures in many countries in the region are insufficient [72]. In contrast to previous studies [25, 26], participants in the present study were still living in countries with high levels of violence, conflict, and human rights abuses, without the possibility to receive help locally. Due to the limited mental health structures and other societal factors (i.e. stigma of mental health problems), the current sample consisted of participants who were actively seeking psychological help through the internet, and may therefore show a higher symptom burden than the general population.

To gain a deeper understanding of the latent classes, we examined individual factors that might impact class membership, with gender emerging as a significant predictor. A large number of studies have shown that women are at greater risk of developing PTSD [e.g. 27]. In line with this, in the present study, female participants were significantly more likely to belong to classes with a high probability of endorsing specific posttraumatic stress symptoms compared to the general low PTSS class. This underlines previous findings that women in conflict-affected areas are more likely to develop a higher severity of posttraumatic stress symptoms compared to men [e.g. 73]. In accordance with previous studies reporting a beneficial effect of social support in maintaining mental health [e.g. 14], social support was a significant predictor of class membership in the present study, insofar as participants with lower social support were more likely to belong to the general high PTSS class, the high PTSS-low avoidance class, or the high dysphoric-low reexperiencing/avoidance class compared to the general low PTSS class. Therefore, it might be suggested that symptom severity increases when social support is lacking. In contrast to the general low PTSS class and the mixed PTSS class, the other classes showed a high probability of endorsing items pertaining to loss of interest, lack of positive emotions, difficulties in concentrating, and sleep disturbances. Thus, it is also possible that the core symptoms of depression (i.e. anhedonia) limit individuals’ social contact or their perception of social support. Concerning trauma-related factors, cumulative trauma, direct exposure to the most distressing trauma, sexual violence during the most distressing trauma, and time since the most distressing trauma consistently emerged as significant predictors. There is strong evidence that the risk of developing PTSD depends on the cumulative exposure to traumatic events in a dose-dependent manner [building block effect; 32, 74]. Indeed, participants who were exposed to a higher number of different traumatic events were more likely to belong to one of the symptomatically burdened classes than to the general low PTSS class. Hence, the likelihood of endorsing more specific posttraumatic stress symptoms—particularly reexperiencing symptoms—seems to increase with the exposure to a greater number of different traumatic events. Exposure to sexual violence during the most distressing event also predicted class membership, insofar as participants who experienced sexual violence during the most distressing event were more likely to belong to the general high PTSS class or the mixed PTSS class than to the general low PTSS class or the high dysphoric-low reexperiencing/avoidance class. In contrast to the latter two classes, the other classes showed a high probability of endorsing intrusive memories and emotional distress due to reminders. This pattern was similar to that found for gender as a predictor of class membership. Although survivors of sexual violence can be of any gender, women seem to experience more sexual violence like rape or sexual assault in conflict situations [73] and appear to react differently to trauma exposure than men, showing more emotional distress due to reminders [75]. Interestingly, participants who experienced the most distressing event in person or as a witness were more likely to belong to the general high PTSS class or the mixed PTSS class than to the general low PTSS class. Previous studies suggest a higher likelihood of developing PTSD from direct trauma exposure than from indirect exposure [76]. Moreover, compared to the general high PTSS class and the mixed PTSS class, participants with direct exposure to the most distressing event were less likely to belong to the high PTSS-low avoidance class. Thus, individuals who had experienced the most distressing event indirectly were less likely to endorse active avoidance while endorsing nearly all other symptoms. Concerning time since the most distressing event, participants who experienced this event less than three years ago were more likely to belong to the general high PTSS or the high PTSS-low avoidance class than to the general low PTSS class. Though not specifically focusing on exposure to the most distressing event, a previous meta-analysis similarly revealed a higher prevalence of PTSD in conflict-affected populations if conflicts were ongoing or had ceased less than a year earlier [8].

### Limitations

Several limitations of the present study should be mentioned. First, all data were assessed online using self-report measures, which may have led to an overestimation of symptom severity. Second, the sample was not drawn from the general Arabic-speaking population originating from the MENA region but rather mostly consisted of young Arabic-speaking individuals, who were predominantly female, highly educated, from urban areas, and had registered for an online treatment to receive help for mental health problems. As such, our findings are restricted to a specific group of people from the region who were already experiencing significant distress and able and willing to seek help online. Thus, it might be possible that the latent classes detected in our help-seeking and highly distressed sample differ from latent classes that may be identified in samples with different ranges of PTSD symptom severity (e.g., in samples randomly drawn from the general population). Third, we did not investigate the frequency, type of exposure, and temporal aspects (like age at the time of exposure, amount of time since exposure) for each specific traumatic event experienced by the participants, and instead focused mainly on the most distressing event in our regression analysis. Finally, as we did not assess participants’ current living conditions, we do not know whether the participants were living in current situations of threat at the time of the study.

### Conclusion

The identification of five different classes, with unique profiles of posttraumatic stress symptoms and differences in comorbid mental health symptoms, highlights the importance of investigating a broad range of posttraumatic stress symptoms as well as comorbid symptomatology that might develop in the aftermath of trauma. Such investigations should help to provide an overall picture of an individual’s impairment and to detect individuals who might be at risk or who need treatment. The identification of classes characterized by a high probability of some symptoms but not of all symptom clusters (i.e. the high PTSS-low avoidance class) implies that there are individuals who experience some posttraumatic stress symptoms but would be unlikely to meet diagnostic criteria for PTSD, as they do not show clinically significant symptoms in all symptom clusters. Nevertheless, these individuals suffer from substantial impairment and show poorer quality of life than participants in the class with a low symptom probability. Moreover, some individuals might be at risk of developing more symptoms or greater symptom severity over time (i.e. the mixed PTSS class). Therefore, the exclusive use of isolated symptoms or diagnoses to decide on the need for treatment might be short-sighted. Furthermore, the identification of specific classes might be useful when deciding which type of treatment fits a specific class best. For example, in patients with chronic PTSD, Burton, Cooper [77] found that membership of a specific latent class (distressed, depressive, or avoidant class) strongly predicted treatment response. In the present sample, the general high PTSS class might benefit from trauma-focused treatments, whereas the high dysphoric-low reexperiencing/avoidance class might benefit more from treatments that focus on behavioral activation or training of social competencies. Future research should investigate whether patients with different posttraumatic stress symptom presentations might benefit differently from specific forms of treatment and whether specific symptom presentations might develop differently over the course of a particular treatment. The overall high level of distress in the current sample highlights the need for specialized treatment for these burdened individuals, taking into account combinations of posttraumatic stress symptoms and symptom severity, comorbid symptoms, specific needs, and resources as well as related predictors. It is worth noting that it is of importance to replicate the results in future studies with other trauma-exposed samples from the MENA region, such as individuals not seeking treatment, in order to draw conclusions about the generalizability of the results.

## Supporting information

S1 Fig Gain in AIC, BIC, aBIC, AWE and LL across models with an increasing number of latent classes. (PDF)

S2 Fig Class-specific item response probability profile plots for symptom endorsement for models with three to seven latent classes. (PDF)

S1 Table Overall symptom frequency and probability of symptom endorsement for each latent class (N = 5,140). PTSS = Posttraumatic stress symptoms; Probability values of endorsement for a specific symptom > 0.70 (high) or < 0.30 (low) in **bold**; SE = Standard Error.
(DOCX)

S2 Table Multinomial logistic regression predicting class membership. PTSS = Posttraumatic stress symptoms; Number of participants reduced to n = 5,110 due to missing data; Categorical predictors were dummy coded; ^a^0 = female, 1 = male; ^b^0 = low education, 1 = high education; ^c^0 = from rural areas, 1 = from urban areas; ^d^0 = low PTI (Political Terror Index), 1 = high PTI (Political Terror Index); ^e^0 = no, 1 = yes; ^f^range from 1 to 24; ^g^0 = more than 3 years, 1 = less than 3 years; SE = Standard Error; CI = Confidence Interval; Significant p values are printed in **bold**.
(DOCX)
