# Supplementary figures and images for: Patterns of posttraumatic stress symptoms, their predictors, and comorbid mental health symptoms in traumatized Arabic-speaking people: A latent class analysis
Source: PLoS One. 2023 Dec 22;18(12):e0295999. doi: 10.1371/journal.pone.0295999 (PMC10745222; doi:10.1371/journal.pone.0295999)

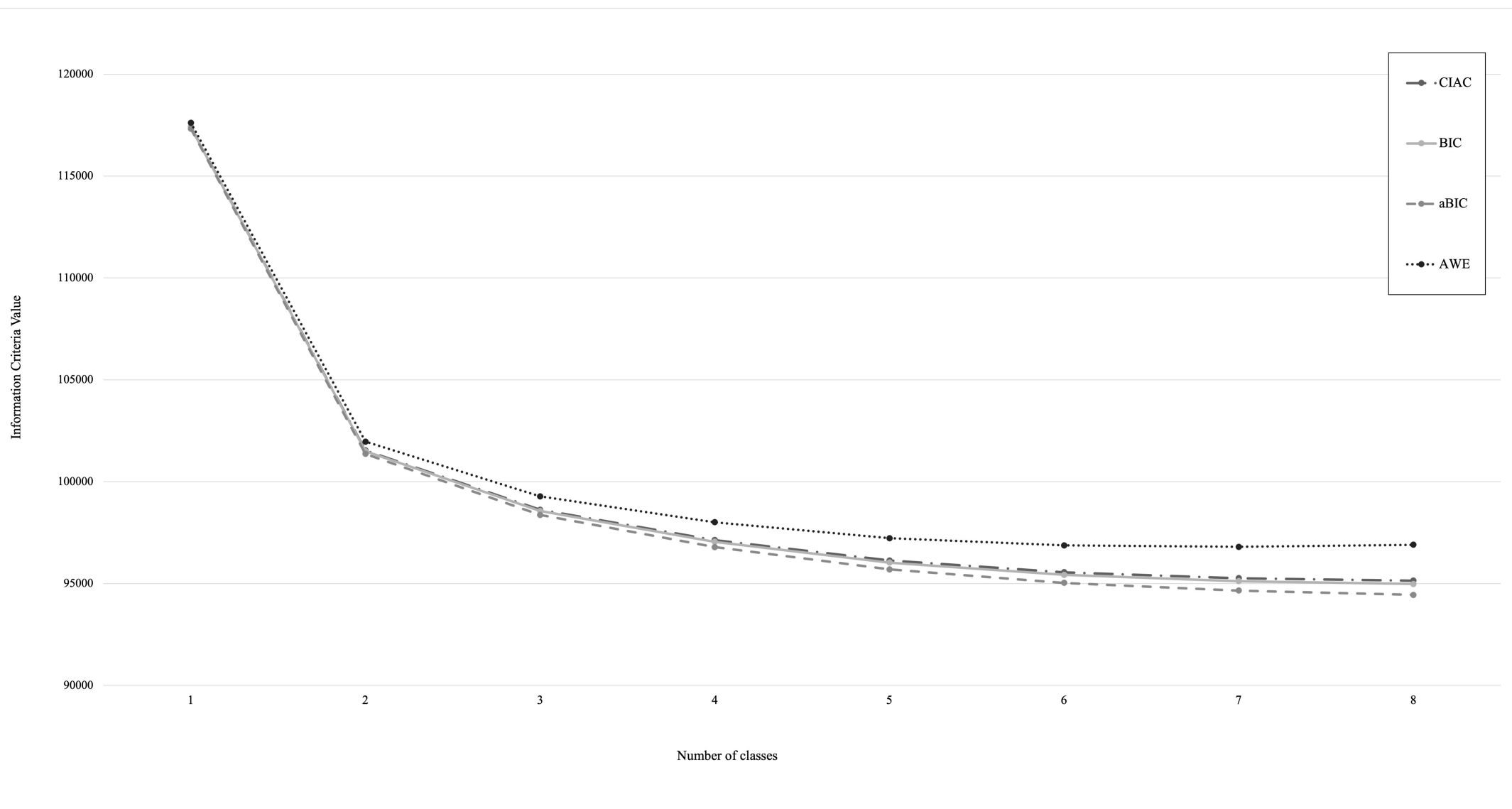

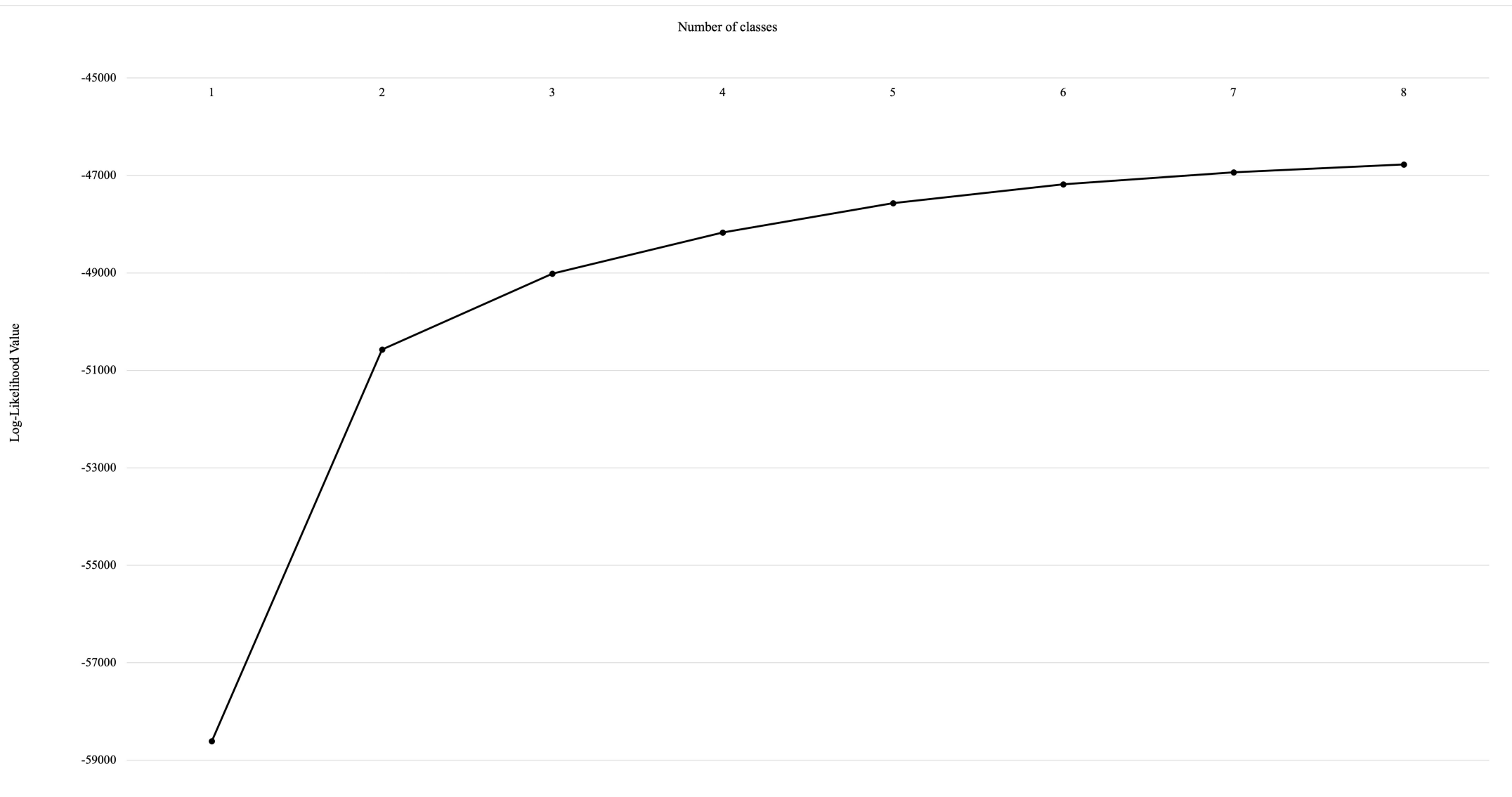

Supplement: S1 Fig — (PDF) [file pone.0295999.s001.pdf]

3-class solution

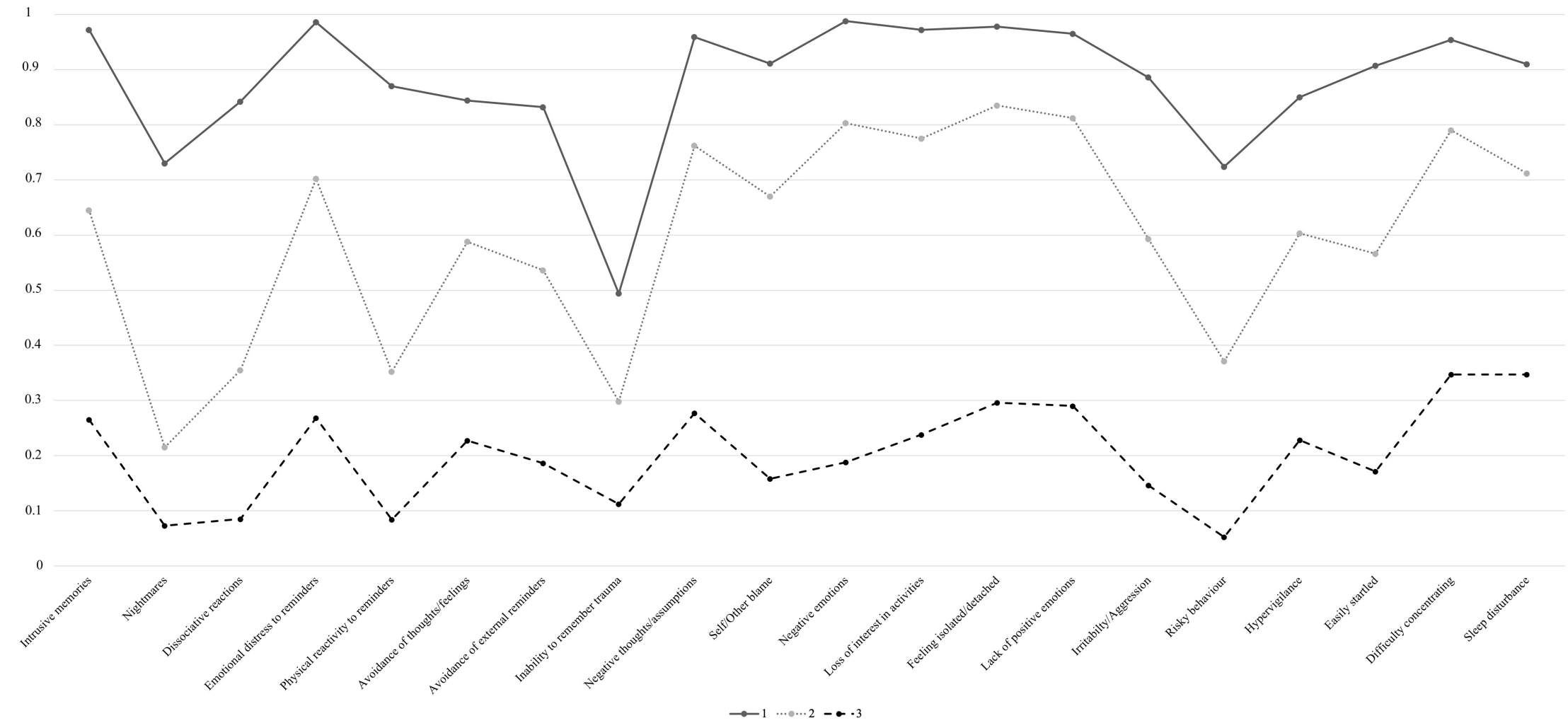

4-class solution

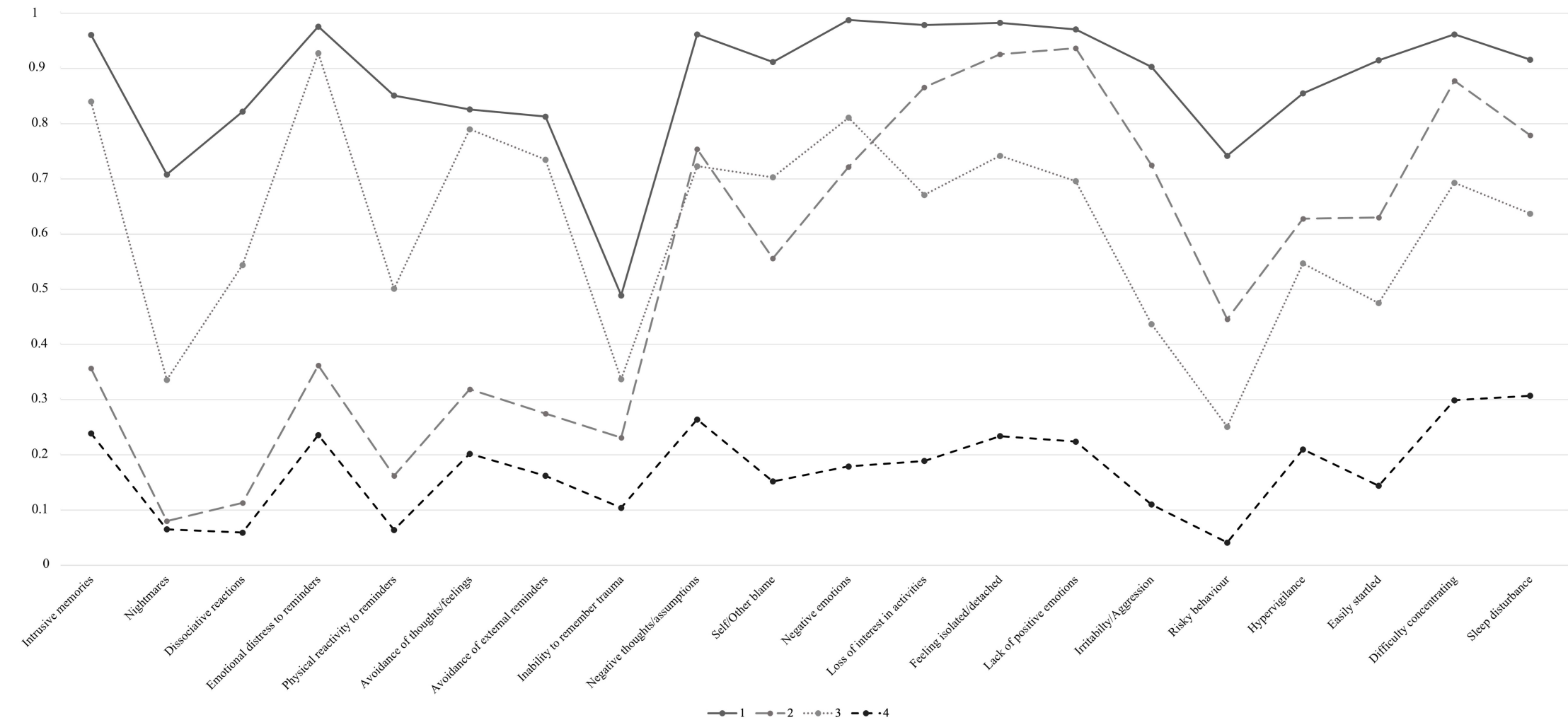

5-class solution

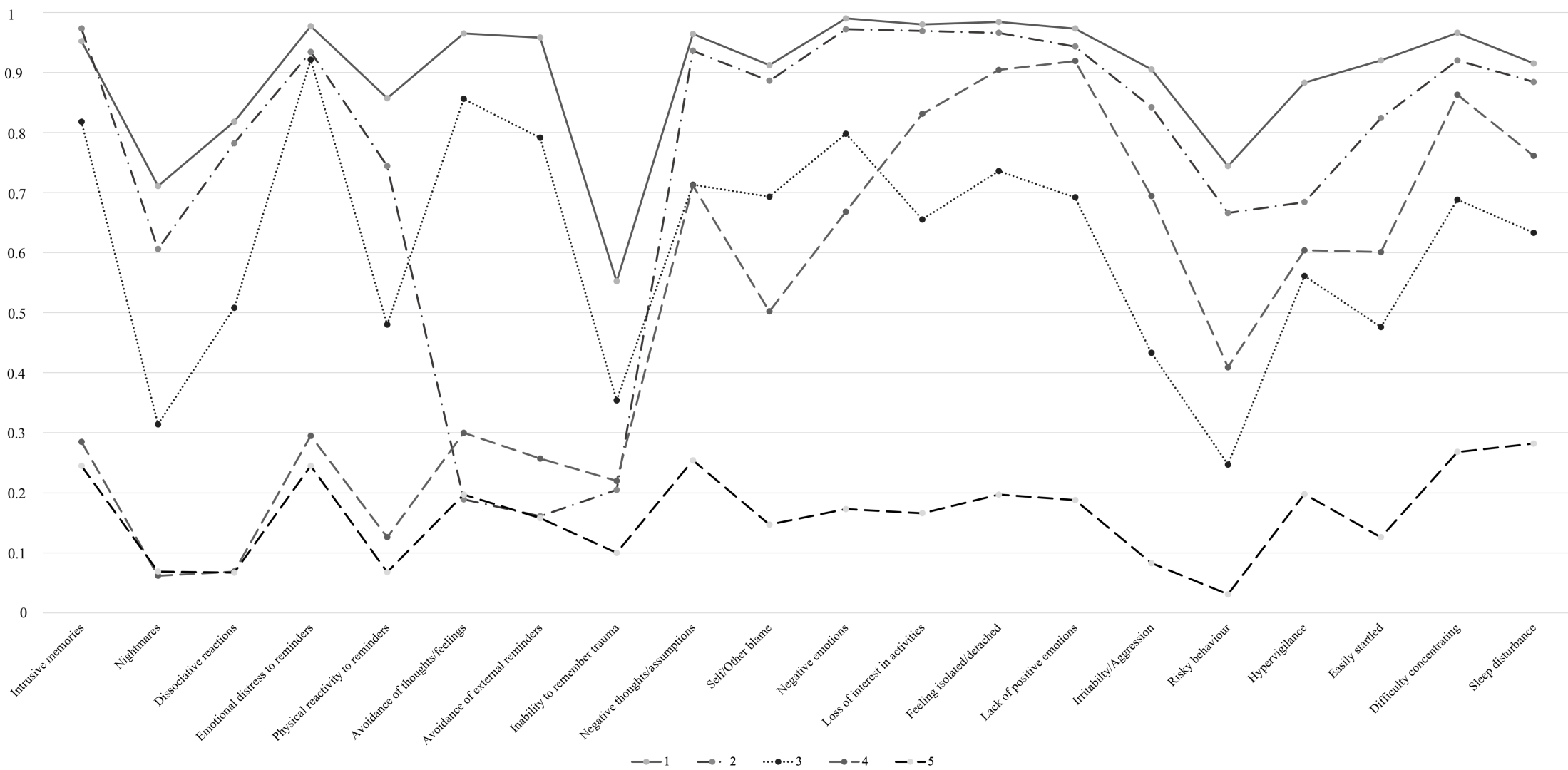

6-class solution

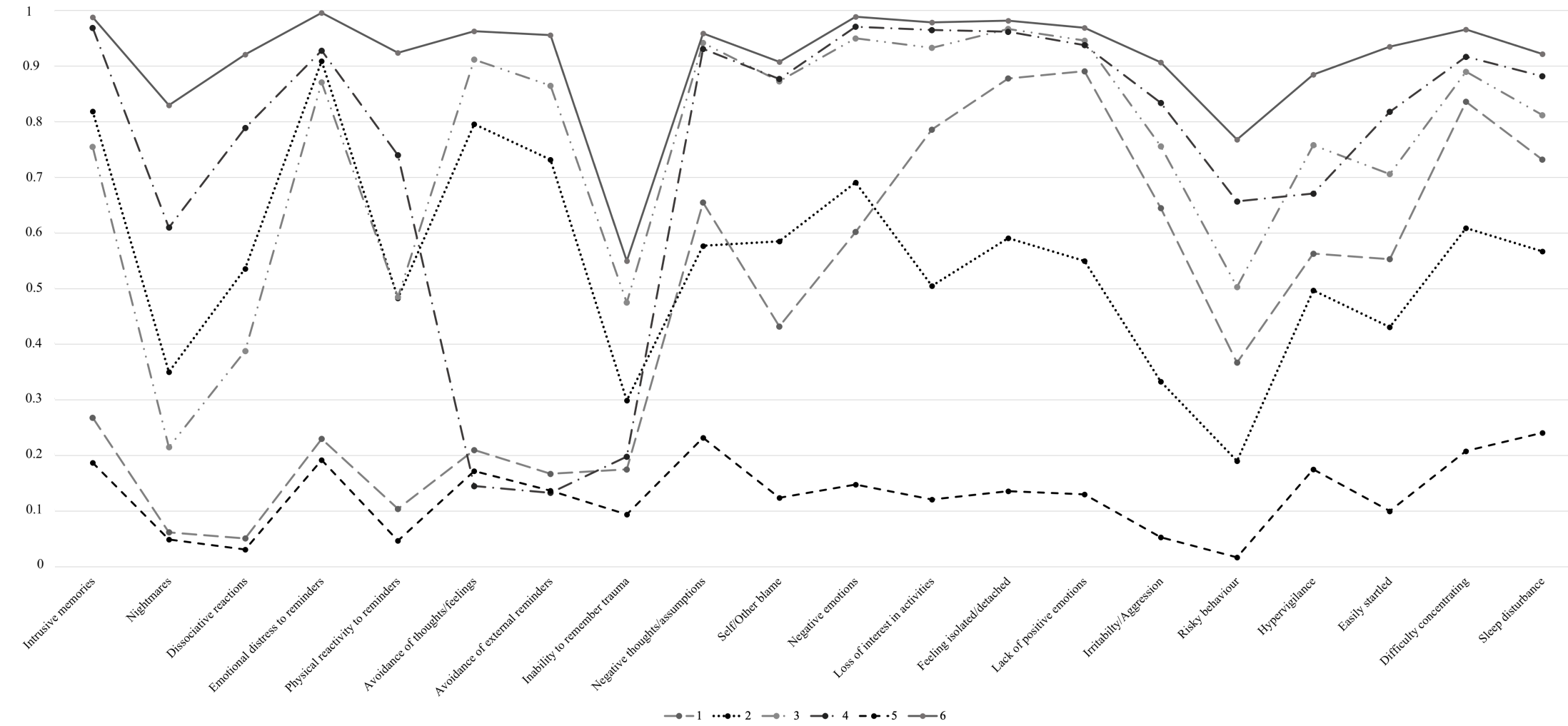

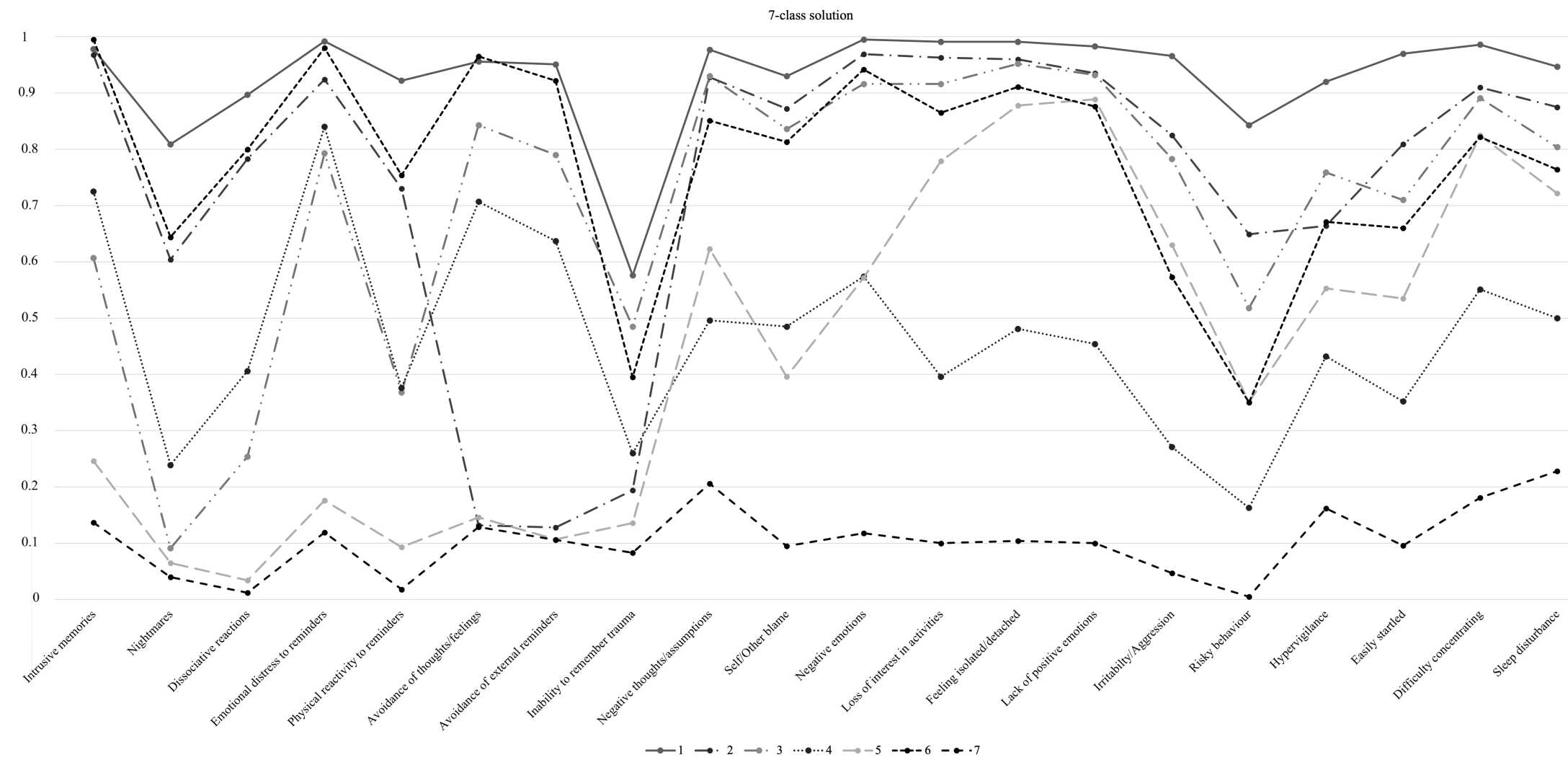

Supplement: S2 Fig — (PDF) [file pone.0295999.s002.pdf]
