# Supplementary material for: Patterns of posttraumatic stress symptoms, their predictors, and comorbid mental health symptoms in traumatized Arabic-speaking people: A latent class analysis
Source: PLoS One. 2023 Dec 22;18(12):e0295999. doi: 10.1371/journal.pone.0295999 (PMC10745222; doi:10.1371/journal.pone.0295999)
Supplement: S1 Table — PTSS = Posttraumatic stress symptoms; Probability values of endorsement for a specific symptom > 0.70 (high) or < 0.30 (low) in bold; SE = Standard Error. (DOCX) [file pone.0295999.s003.docx]

| Posttraumatic stress symptoms | General high PTSS  class  (n = 2,250) | | High PTSS-low  avoidance class  (n = 660) | | Mixed PTSS class  (n = 1,076) | | High dysphoric-low reexperiencing/ avoidance class  (n = 721) | | General low PTSS  class (n = 433) | |
| --- | --- | --- | --- | --- | --- | --- | --- | --- | --- | --- |
|  | Probability  of  endorsement | SE | Probability  of  endorsement | SE | Probability  of  endorsement | SE | Probability  of  endorsement | SE | Probability  of  endorsement | SE |
| Intrusive memories | **0.952** | 0.006 | **0.973** | 0.012 | **0.818** | 0.022 | **0.285** | 0.031 | **0.245** | 0.031 |
| Nightmares | **0.711** | 0.014 | 0.606 | 0.030 | 0.314 | 0.023 | **0.062** | 0.012 | **0.069** | 0.016 |
| Dissociative reactions | **0.818** | 0.011 | **0.782** | 0.025 | 0.508 | 0.027 | **0.069** | 0.018 | **0.067** | 0.020 |
| Emotional distress due to reminders | **0.977** | 0.004 | **0.934** | 0.014 | **0.921** | 0.014 | **0.295** | 0.051 | **0.245** | 0.033 |
| Physical reactivity to reminders | **0.857** | 0.011 | **0.744** | 0.028 | 0.480 | 0.025 | **0.126** | 0.021 | **0.068** | 0.016 |
| Avoidance of thoughts/feelings | **0.965** | 0.007 | **0.189** | 0.034 | **0.856** | 0.022 | 0.300 | 0.051 | **0.197** | 0.026 |
| Avoidance of external reminders | **0.958** | 0.008 | **0.161** | 0.030 | **0.791** | 0.024 | **0.257** | 0.046 | **0.158** | 0.023 |
| Inability to remember trauma | 0.552 | 0.012 | **0.205** | 0.019 | 0.354 | 0.019 | **0.220** | 0.028 | **0.100** | 0.016 |
| Negative thoughts/assumptions | **0.964** | 0.005 | **0.936** | 0.012 | **0.713** | 0.027 | **0.712** | 0.039 | **0.254** | 0.027 |
| Self/Other blame | **0.912** | 0.007 | **0.886** | 0.016 | 0.693 | 0.023 | 0.502 | 0.045 | **0.147** | 0.023 |
| Negative emotions | **0.990** | 0.003 | **0.972** | 0.009 | **0.798** | 0.022 | 0.668 | 0.043 | **0.173** | 0.024 |
| Loss of interest in activities | **0.980** | 0.004 | **0.969** | 0.009 | 0.655 | 0.033 | **0.831** | 0.030 | **0.166** | 0.035 |
| Feeling isolated/detached | **0.984** | 0.003 | **0.966** | 0.009 | **0.736** | 0.029 | **0.904** | 0.020 | **0.197** | 0.048 |
| Lack of positive emotions | **0.973** | 0.004 | **0.943** | 0.012 | 0.692 | 0.031 | **0.919** | 0.021 | **0.188** | 0.044 |
| Irritabilty/aggression | **0.905** | 0.010 | **0.842** | 0.020 | 0.433 | 0.032 | 0.694 | 0.032 | **0.083** | 0.028 |
| Risky behavior | **0.744** | 0.014 | 0.666 | 0.025 | **0.247** | 0.023 | 0.409 | 0.031 | **0.031** | 0.014 |
| Hypervigilance | **0.883** | 0.009 | 0.684 | 0.024 | 0.561 | 0.025 | 0.604 | 0.033 | **0.198** | 0.024 |
| Easily startled | **0.920** | 0.009 | **0.824** | 0.023 | 0.476 | 0.026 | 0.601 | 0.032 | **0.126** | 0.026 |
| Difficulty concentrating | **0.966** | 0.005 | **0.920** | 0.014 | 0.688 | 0.024 | **0.863** | 0.019 | **0.268** | 0.043 |
| Sleep disturbances | **0.915** | 0.008 | **0.884** | 0.016 | 0.633 | 0.023 | **0.761** | 0.022 | **0.282** | 0.037 |
