# Supplementary material for: Patterns of posttraumatic stress symptoms, their predictors, and comorbid mental health symptoms in traumatized Arabic-speaking people: A latent class analysis
Source: PLoS One. 2023 Dec 22;18(12):e0295999. doi: 10.1371/journal.pone.0295999 (PMC10745222; doi:10.1371/journal.pone.0295999)
Supplement: S2 Table — PTSS = Posttraumatic stress symptoms; Number of participants reduced to n = 5,110 due to missing data; Categorical predictors were dummy coded; a0 = female, 1 = male; b0 = low education, 1 = high education; c0 = from rural areas, 1 = from urban areas; d0 = low PTI (Political Terror Index), 1 = high PTI (Political Terror Index); e0 = no, 1 = yes; frange from 1 to 24; g0 = more than 3 years, 1 = less than 3 years; SE = Standard Error; CI = Confidence Interval; Significant p values are printed in bold. (DOCX) [file pone.0295999.s004.docx]

| Class comparison | Estimate | SE | Odds ratio | 95% CI | | p |
| --- | --- | --- | --- | --- | --- | --- |
|  |  |  |  | lower | upper |  |
| General high vs. General low PTSS class (reference) |  |  |  |  |  |  |
| Age | -0.01 | 0.01 | 0.99 | 0.98 | 1.01 | 0.415 |
| Gender^a^ | -0.61 | 0.14 | 0.54 | 0.42 | 0.71 | **<0.001** |
| Education^b^ | -0.06 | 0.21 | 0.95 | 0.63 | 1.41 | 0.788 |
| Place of residence^c^ | 0.30 | 0.18 | 1.35 | 0.95 | 1.91 | 0.093 |
| PTI country of origin^d^ | -0.47 | 0.26 | 0.62 | 0.37 | 1.04 | 0.071 |
| PTI country of residence^d^ | 0.23 | 0.24 | 1.26 | 0.78 | 2.01 | 0.341 |
| Flight experience^e^ | 0.31 | 0.30 | 1.36 | 0.76 | 2.43 | 0.296 |
| Social support | -0.03 | 0.00 | 0.97 | 0.97 | 0.98 | **<0.001** |
| Cumulative traumatic events^f^ | 0.29 | 0.03 | 1.34 | 1.26 | 1.42 | **<0.001** |
| Sexual violence during most distressing trauma^e^ | 0.88 | 0.20 | 2.41 | 1.62 | 3.58 | **<0.001** |
| Direct exposure to most distressing trauma^e^ | 0.41 | 0.14 | 1.51 | 1.15 | 1.98 | **0.003** |
| Danger to life during most distressing trauma^e^ | 0.03 | 0.12 | 1.03 | 0.82 | 1.31 | 0.785 |
| Time since most distressing trauma^g^ | 0.60 | 0.12 | 1.81 | 1.42 | 2.31 | **<0.001** |
| High PTSS-low avoidance vs. General low PTSS class (reference) |  |  |  |  |  |  |
| Age | 0.01 | 0.01 | 1.01 | 0.99 | 1.03 | 0.444 |
| Gender^a^ | -0.48 | 0.17 | 0.62 | 0.45 | 0.87 | **0.005** |
| Education^b^ | -0.26 | 0.24 | 0.77 | 0.48 | 1.23 | 0.271 |
| Place of residence^c^ | -0.14 | 0.21 | 0.87 | 0.58 | 1.31 | 0.501 |
| PTI country of origin^d^ | -0.50 | 0.31 | 0.61 | 0.33 | 1.11 | 0.104 |
| PTI country of residence^d^ | 0.47 | 0.29 | 1.60 | 0.91 | 2.79 | 0.100 |
| Flight experience^e^ | 0.21 | 0.35 | 1.23 | 0.62 | 2.42 | 0.551 |
| Social support | -0.03 | 0.00 | 0.97 | 0.96 | 0.98 | **<0.001** |
| Cumulative traumatic events^f^ | 0.29 | 0.03 | 1.33 | 1.25 | 1.42 | **<0.001** |
| Sexual violence during most distressing trauma^e^ | 0.51 | 0.23 | 1.66 | 1.05 | 2.62 | 0.029 |
| Direct exposure to most distressing trauma^e^ | -0.01 | 0.17 | 0.99 | 0.72 | 1.37 | 0.971 |
| Danger to life during most distressing trauma^e^ | -0.04 | 0.15 | 0.96 | 0.71 | 1.28 | 0.766 |
| Time since most distressing trauma^g^ | 0.94 | 0.15 | 2.57 | 1.92 | 3.45 | **<0.001** |
| Mixed vs. General low PTSS class (reference) |  |  |  |  |  |  |
| Age | -0.02 | 0.01 | 0.98 | 0.96 | 1.00 | 0.060 |
| Gender^a^ | -0.61 | 0.15 | 0.54 | 0.40 | 0.74 | **<0.001** |
| Education^b^ | -0.06 | 0.22 | 0.94 | 0.61 | 1.46 | 0.796 |
| Place of residence^c^ | 0.34 | 0.21 | 1.41 | 0.94 | 2.11 | 0.099 |
| PTI country of origin^d^ | -0.39 | 0.30 | 0.68 | 0.38 | 1.21 | 0.188 |
| PTI country of residence^d^ | 0.27 | 0.27 | 1.30 | 0.76 | 2.23 | 0.334 |
| Flight experience^e^ | 0.60 | 0.31 | 1.81 | 0.99 | 3.31 | 0.052 |
| Social support | -0.01 | 0.00 | 0.99 | 0.99 | 1.00 | 0.056 |
| Cumulative traumatic events^f^ | 0.18 | 0.03 | 1.19 | 1.12 | 1.27 | **<0.001** |
| Sexual violence during most distressing trauma^e^ | 0.76 | 0.21 | 2.14 | 1.41 | 3.26 | **<0.001** |
| Direct exposure to most distressing trauma^e^ | 0.51 | 0.16 | 1.66 | 1.22 | 2.27 | **0.001** |
| Danger to life during most distressing trauma^e^ | -0.22 | 0.13 | 0.80 | 0.62 | 1.04 | 0.099 |
| Time since most distressing trauma^g^ | 0.23 | 0.14 | 1.26 | 0.97 | 1.65 | 0.088 |
| High dysphoric-low reexperiencing/avoidance vs. General low PTSS class (reference) |  |  |  |  |  |  |
| Age | 0.00 | 0.01 | 1.00 | 0.98 | 1.02 | 0.969 |
| Gender^a^ | 0.06 | 0.15 | 1.06 | 0.79 | 1.43 | 0.701 |
| Education^b^ | -0.11 | 0.24 | 0.89 | 0.56 | 1.43 | 0.637 |
| Place of residence^c^ | 0.08 | 0.21 | 1.08 | 0.71 | 1.63 | 0.722 |
| PTI country of origin^d^ | -0.08 | 0.31 | 0.92 | 0.50 | 1.69 | 0.786 |
| PTI country of residence^d^ | 0.18 | 0.28 | 1.20 | 0.69 | 2.07 | 0.522 |
| Flight experience^e^ | 0.63 | 0.33 | 1.88 | 0.99 | 3.56 | 0.054 |
| Social support | -0.03 | 0.00 | 0.97 | 0.97 | 0.98 | **<0.001** |
| Cumulative traumatic events^f^ | 0.11 | 0.04 | 1.12 | 1.04 | 1.21 | **0.002** |
| Sexual violence during most distressing trauma^e^ | 0.11 | 0.25 | 1.12 | 0.69 | 1.81 | 0.659 |
| Direct exposure to most distressing trauma^e^ | 0.23 | 0.17 | 1.26 | 0.91 | 1.74 | 0.163 |
| Danger to life during most distressing trauma^e^ | -0.03 | 0.15 | 0.97 | 0.73 | 1.29 | 0.857 |
| Time since most distressing trauma^g^ | -0.12 | 0.15 | 0.89 | 0.66 | 1.19 | 0.437 |
| High PTSS-low avoidance vs. General high PTSS class (reference) |  |  |  |  |  |  |
| Age | 0.02 | 0.01 | 1.02 | 1.00 | 1.03 | 0.090 |
| Gender^a^ | 0.13 | 0.14 | 1.14 | 0.87 | 1.51 | 0.341 |
| Education^b^ | -0.21 | 0.16 | 0.81 | 0.59 | 1.12 | 0.208 |
| Place of residence^c^ | -0.44 | 0.16 | 0.65 | 0.47 | 0.89 | 0.008 |
| PTI country of origin^d^ | -0.03 | 0.21 | 0.98 | 0.65 | 1.47 | 0.906 |
| PTI country of residence^d^ | 0.24 | 0.20 | 1.27 | 0.86 | 1.88 | 0.227 |
| Flight experience^e^ | -0.10 | 0.23 | 0.90 | 0.57 | 1.42 | 0.657 |
| Social support | 0.00 | 0.00 | 1.00 | 0.99 | 1.00 | 0.532 |
| Cumulative traumatic events^f^ | -0.01 | 0.02 | 0.99 | 0.96 | 1.03 | 0.698 |
| Sexual violence during most distressing trauma^e^ | -0.37 | 0.15 | 0.69 | 0.52 | 0.92 | 0.012 |
| Direct exposure to most distressing trauma^e^ | -0.42 | 0.13 | 0.66 | 0.51 | 0.85 | **0.002** |
| Danger to life during most distressing trauma^e^ | -0.08 | 0.12 | 0.93 | 0.74 | 1.16 | 0.505 |
| Time since most distressing trauma^g^ | 0.35 | 0.12 | 1.42 | 1.13 | 1.78 | **0.003** |
| Mixed vs. General high PTSS class (reference) |  |  |  |  |  |  |
| Age | -0.01 | 0.01 | 0.99 | 0.97 | 1.00 | 0.141 |
| Gender^a^ | 0.00 | 0.13 | 1.00 | 0.78 | 1.28 | 0.983 |
| Education^b^ | 0.00 | 0.15 | 1.00 | 0.74 | 1.34 | 0.985 |
| Place of residence^c^ | 0.04 | 0.16 | 1.04 | 0.76 | 1.43 | 0.785 |
| PTI country of origin^d^ | 0.08 | 0.19 | 1.09 | 0.75 | 1.57 | 0.660 |
| PTI country of residence^d^ | 0.04 | 0.18 | 1.04 | 0.73 | 1.47 | 0.839 |
| Flight experience^e^ | 0.29 | 0.18 | 1.33 | 0.93 | 1.90 | 0.113 |
| Social support | 0.02 | 0.00 | 1.02 | 1.01 | 1.03 | **<0.001** |
| Cumulative traumatic events^f^ | -0.12 | 0.02 | 0.89 | 0.86 | 0.92 | **<0.001** |
| Sexual violence during most distressing trauma^e^ | -0.12 | 0.12 | 0.89 | 0.71 | 1.12 | 0.311 |
| Direct exposure to most distressing trauma^e^ | 0.09 | 0.13 | 1.10 | 0.85 | 1.41 | 0.471 |
| Danger to life during most distressing trauma^e^ | -0.25 | 0.10 | 0.78 | 0.64 | 0.94 | 0.008 |
| Time since most distressing trauma^g^ | -0.36 | 0.10 | 0.70 | 0.57 | 0.84 | **<0.001** |
| High dysphoric-low reexperiencing/avoidance vs. General high PTSS class (reference) |  |  |  |  |  |  |
| Age | 0.01 | 0.01 | 1.01 | 0.99 | 1.02 | 0.349 |
| Gender^a^ | 0.67 | 0.12 | 1.95 | 1.55 | 2.46 | **<0.001** |
| Education^b^ | -0.06 | 0.16 | 0.94 | 0.69 | 1.30 | 0.722 |
| Place of residence^c^ | -0.22 | 0.16 | 0.80 | 0.59 | 1.09 | 0.153 |
| PTI country of origin^d^ | 0.39 | 0.20 | 1.47 | 0.99 | 2.20 | 0.057 |
| PTI country of residence^d^ | -0.05 | 0.19 | 0.95 | 0.66 | 1.37 | 0.790 |
| Flight experience^e^ | 0.32 | 0.20 | 1.38 | 0.93 | 2.04 | 0.108 |
| Social support | 0.00 | 0.00 | 1.00 | 1.00 | 1.01 | 0.593 |
| Cumulative traumatic events^f^ | -0.18 | 0.02 | 0.84 | 0.80 | 0.87 | **<0.001** |
| Sexual violence during most distressing trauma^e^ | -0.77 | 0.15 | 0.46 | 0.34 | 0.63 | **<0.001** |
| Direct exposure to most distressing trauma^e^ | -0.18 | 0.13 | 0.83 | 0.65 | 1.07 | 0.153 |
| Danger to life during most distressing trauma^e^ | -0.06 | 0.11 | 0.94 | 0.77 | 1.16 | 0.576 |
| Time since most distressing trauma^g^ | -0.71 | 0.11 | 0.49 | 0.39 | 0.61 | **<0.001** |
| Mixed vs. High PTSS-low avoidance class (reference) |  |  |  |  |  |  |
| Age | -0.03 | 0.01 | 0.97 | 0.95 | 0.99 | 0.011 |
| Gender^a^ | -0.13 | 0.17 | 0.88 | 0.63 | 1.21 | 0.425 |
| Education^b^ | 0.20 | 0.20 | 1.22 | 0.83 | 1.80 | 0.302 |
| Place of residence^c^ | 0.48 | 0.20 | 1.62 | 1.10 | 2.39 | 0.015 |
| PTI country of origin^d^ | 0.11 | 0.26 | 1.11 | 0.67 | 1.84 | 0.674 |
| PTI country of residence^d^ | -0.21 | 0.25 | 0.81 | 0.50 | 1.32 | 0.403 |
| Flight experience^e^ | 0.39 | 0.26 | 1.48 | 0.88 | 2.46 | 0.136 |
| Social support | 0.02 | 0.00 | 1.02 | 1.01 | 1.03 | **<0.001** |
| Cumulative traumatic events^f^ | -0.11 | 0.02 | 0.90 | 0.86 | 0.93 | **<0.001** |
| Sexual violence during most distressing trauma^e^ | 0.26 | 0.17 | 1.29 | 0.93 | 1.79 | 0.128 |
| Direct exposure to most distressing trauma^e^ | 0.51 | 0.16 | 1.67 | 1.22 | 2.29 | **0.001** |
| Danger to life during most distressing trauma^e^ | -0.18 | 0.13 | 0.84 | 0.65 | 1.09 | 0.180 |
| Time since most distressing trauma^g^ | -0.71 | 0.13 | 0.49 | 0.38 | 0.64 | **<0.001** |
| High dysphoric-low reexperiencing/avoidance vs. High PTSS-low avoidance class (reference) |  |  |  |  |  |  |
| Age | -0.01 | 0.01 | 0.99 | 0.97 | 1.01 | 0.384 |
| Gender^a^ | 0.54 | 0.16 | 1.71 | 1.25 | 2.34 | **0.001** |
| Education^b^ | 0.15 | 0.21 | 1.16 | 0.77 | 1.74 | 0.480 |
| Place of residence^c^ | 0.22 | 0.20 | 1.24 | 0.85 | 1.82 | 0.271 |
| PTI country of origin^d^ | 0.41 | 0.27 | 1.51 | 0.89 | 2.56 | 0.124 |
| PTI country of residence^d^ | -0.29 | 0.25 | 0.75 | 0.46 | 1.22 | 0.245 |
| Flight experience^e^ | 0.42 | 0.28 | 1.53 | 0.89 | 2.63 | 0.126 |
| Social support | 0.00 | 0.00 | 1.00 | 1.00 | 1.01 | 0.356 |
| Cumulative traumatic events^f^ | -0.17 | 0.03 | 0.84 | 0.80 | 0.89 | **<0.001** |
| Sexual violence during most distressing trauma^e^ | -0.40 | 0.20 | 0.67 | 0.45 | 0.99 | 0.045 |
| Direct exposure to most distressing trauma^e^ | 0.24 | 0.16 | 1.27 | 0.92 | 1.74 | 0.144 |
| Danger to life during most distressing trauma^e^ | 0.02 | 0.14 | 1.02 | 0.77 | 1.34 | 0.897 |
| Time since most distressing trauma^g^ | -1.06 | 0.14 | 0.35 | 0.26 | 0.46 | **<0.001** |
| High dysphoric-low reexperiencing/avoidance vs. Mixed PTSS class (reference) |  |  |  |  |  |  |
| Age | 0.02 | 0.01 | 1.02 | 1.00 | 1.04 | 0.045 |
| Gender^a^ | 0.67 | 0.15 | 1.95 | 1.46 | 2.60 | **<0.001** |
| Education^b^ | -0.06 | 0.20 | 0.95 | 0.64 | 1.40 | 0.780 |
| Place of residence^c^ | -0.27 | 0.20 | 0.77 | 0.52 | 1.13 | 0.181 |
| PTI country of origin^d^ | 0.31 | 0.26 | 1.36 | 0.82 | 2.24 | 0.232 |
| PTI country of residence^d^ | -0.09 | 0.24 | 0.92 | 0.58 | 1.46 | 0.716 |
| Flight experience^e^ | 0.04 | 0.23 | 1.04 | 0.65 | 1.64 | 0.882 |
| Social support | -0.02 | 0.00 | 0.98 | 0.97 | 0.99 | **<0.001** |
| Cumulative traumatic events^f^ | -0.06 | 0.03 | 0.94 | 0.89 | 0.99 | 0.019 |
| Sexual violence during most distressing trauma^e^ | -0.65 | 0.18 | 0.52 | 0.36 | 0.74 | <**0.001** |
| Direct exposure to most distressing trauma^e^ | -0.28 | 0.16 | 0.76 | 0.55 | 1.04 | 0.085 |
| Danger to life during most distressing trauma^e^ | 0.20 | 0.13 | 1.22 | 0.95 | 1.56 | 0.124 |
| Time since most distressing trauma^g^ | -0.35 | 0.13 | 0.70 | 0.54 | 0.91 | 0.008 |
